# Chimeric Avidin – NMR Structure and Dynamics of a 56 kDa Homotetrameric Thermostable Protein

**DOI:** 10.1371/journal.pone.0100564

**Published:** 2014-06-24

**Authors:** Helena Tossavainen, Sampo Kukkurainen, Juha A. E. Määttä, Niklas Kähkönen, Tero Pihlajamaa, Vesa P. Hytönen, Markku S. Kulomaa, Perttu Permi

**Affiliations:** 1 Program in Structural Biology and Biophysics, Institute of Biotechnology, University of Helsinki, Helsinki, Finland; 2 Institute of Biomedical Technology, University of Tampere and Tampere University Hospital, Tampere, Finland; 3 BioMediTech, Tampere, Finland; 4 Fimlab Laboratories, Tampere, Finland; Spanish National Cancer Center, Spain

## Abstract

Chimeric avidin (ChiAVD) is a product of rational protein engineering remarkably resistant to heat and harsh conditions. In quest of the fundamentals behind factors affecting stability we have elucidated the solution NMR spectroscopic structure of the biotin–bound form of ChiAVD and characterized the protein dynamics through ^15^N relaxation and hydrogen/deuterium (H/D) exchange of this and the biotin–free form. To surmount the challenges arising from the very large size of the protein for NMR spectroscopy, we took advantage of its high thermostability. Conventional triple resonance experiments for fully protonated proteins combined with methyl–detection optimized experiments acquired at 58°C were adequate for the structure determination of this 56 kDa protein. The model–free parameters derived from the ^15^N relaxation data reveal a remarkably rigid protein at 58°C in both the biotin–bound and the free forms. The H/D exchange experiments indicate a notable increase in hydrogen protection upon biotin binding.

## Introduction

Chicken egg–white avidin and its bacterial analogue streptavidin from *Streptomyces avidinii* bind their natural ligand biotin with an extremely high affinity (dissociation constant *K_d_*∼10^−15^ M). In addition, they are remarkably stable against heat and harsh conditions such as proteolysis, denaturants and extremes of pH. These exceptional properties are widely employed in (strept)avidin biotechnological applications which typically rely on bridging a biotinylated target molecule binder to (strept)avidin [1], [2] often in solution conditions very unnatural to proteins. Chemical and genetical engineering of avidin and streptavidin have further extended the diversity of the techniques [3].

**Figure 1:**
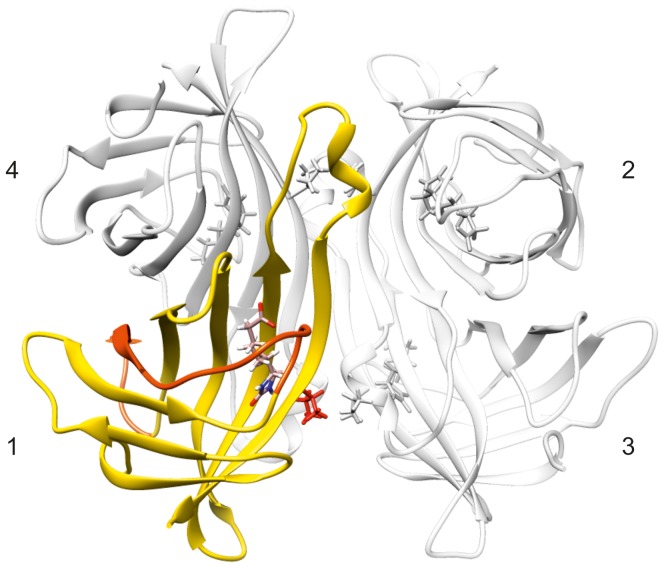
The molecular structure of avidin family of proteins. The structure, here represented with biotin-bound avidin (PDB identifier 2AVI), is a homotetramer composed of units of ∼128 residues. Subunits are numbered according to [4]. Each subunit binds one biotin molecule, shown in stick representation. ChiAVD is a hybrid of avidin and AVR4, in which the segment highlighted in orange in avidin (residues 38–60, 23 residues) is replaced by the sequentially related segment found in AVR4 (residues 38–58, 21 residues). Also, Ile 117 of avidin, shown in red, is replaced by a tyrosine found in AVR4.

Despite low sequence similarity, proteins of the avidin family have a remarkably similar molecular structure composed of four identical subunits (of 128 residues in avidin, Figure 1). The monomeric unit consists of an antiparallel eight–stranded β barrel each of which accommodates one biotin molecule at one end of the barrel. The four avidin subunits are arranged in a dimer of dimers [4]. This quaternary structure results in three distinct interfaces: the 1–4 interface is characterized by hydrophobic and polar interactions of such extent that the dimer can be considered as a single structural unit whereas the 1–3 interface is the weakest, composed only of three residues in avidin. The 1–2 interface is important for the tetramer stability and biotin binding affinity. There, a crucial tryptophan residue interacts with biotin bound in the adjacent subunit [4], [5]. Excitingly, the structural similarity encompasses also the biotin–free forms of the proteins. The binding site is preformed in the free form, and no significant tertiary or quaternary structure rearrangements are needed in order to achieve the tight protein–ligand interaction. The stability of the free form is, however, markedly lower than that of the bound form. This is reflected in lower unfolding and oligomer dissociation temperatures [6].

ChiAVD(I117Y), hereafter referred to as ChiAVD, is a product of rational protein engineering [7], [8]. It is a hyperthermostable hybrid of avidin and avidin-related protein 4 (AVR4) [9]–[11] obtained by replacing a 23–residue segment in avidin with the corresponding segment found in AVR4 and additionally introducing an Ile to Tyr, π–π,1–3 interface–stabilizing point mutation. ChiAVD is remarkably resistant to heat with a transition midpoint temperature, *T_m_*, for thermal unfolding of 111.1°C in the free and ∼130°C in the bound form. In the presence of SDS, it dissociates into monomers only at ∼95°C and 110°C in the free and bound form, respectively. ChiAVD is the most thermostable avidin studied to date. It is also resistant to harsh conditions such as extremes of pH and various organic solvents, even at high temperature. The biotin–binding properties of ChiAVD are comparable to those of AVR4 [7]. Since ChiAVD has successfully been applied in novel approaches in biotechnology [12], [13], the understanding of the molecular properties of the protein is of our special interest. The structure of ChiAVD in the biotin–free form has recently been solved by X–ray crystallography [8]. In this study we present the solution NMR structure of ChiAVD in the biotin–bound form determined at 58°C. Remarkably, the structure of this 56 kDa protein was solved from a uniformly ^13^C/^15^N–labelled sample using triple resonance experiments designed for fully protonated samples together with a set of experiments optimized for detection of methyl–containing residues [14]. The work stands out as a cost–effective approach for the structure determination of large proteins via combination of an optimized measurement temperature with experiments for efficient assignment of residues serving long–range NOEs.

NMR is unique in providing access to residue–specific protein dynamics on a wide time scale [15]. Here we explore the backbone motions of the free and bound ChiAVD through ^15^N relaxation and hydrogen/deuterium (H/D) exchange experiments. The order parameters, S^2^, derived from the relaxation data, reporting on the backbone nano– to picosecond motion, reveal a remarkably stable protein at 58°C in both the free and biotin–bound forms. The H/D exchange experiments indicate a notable increase in hydrogen protection upon biotin binding.

## Materials and Methods

### NMR spectroscopy, experiments and data analysis

Expression and purification of the ^13^C/^15^N–labelled ChiAVD as well as resonance assignment have been described previously [16]. Spectra for structure determination were acquired with a Varian INOVA 800 MHz spectrometer equipped with a cryogenic probehead, at 58°C. The spectra were processed with Vnmr 6.1C (Varian Inc.) and analysed with Sparky 3.110 (T. D. Goddard and D. G. Kneller, University of California, San Francisco).

Distance restraints were obtained from NOE peaks picked from ^13^C, ^1^H NOESY–HSQC, and ^15^N, ^1^H NOESY–HSQC spectra acquired from a sample dissolved in 92/8% H_2_O/D_2_O and ^13^C, ^1^H HSQC–NOESY acquired from a sample in 100% D_2_O, the latter being especially useful for NOEs arising from methyl groups. By inspection of available avidin crystal structures in the RCSB protein data bank (PDB), intersubunit (1–2 and 1–4) NOE peaks were identified and manually assigned. With Cyana [17] version 2.1, two hundred 1–4 dimer structures were calculated with automatic assignment carried out for the intrasubunit peaks, and manually assigned NOE peak lists for 1–4 intersubunit restraints. In addition to distance restraints, H–bond (from H/D exchange experiments), θ/ψ restraints from TALOS [18], χ^1^ restraints deduced from *J*(C–C′) and *J*(C–N)–coupling spectra and ^1^H–^15^N residual dipolar couplings (RDCs) from spectra acquired from a sample in dilute solution of bicelles at 40°C (see next subheading) were used. Twenty structures with the lowest target function were selected. An initial tetrameric structure was built by duplicating a dimer structure and positioning the two dimers at an approximately correct orientation. From each starting tetramer a set of 10 structures was calculated with XPLOR–NIH [19] version 2.29 using all the available restraints. Of the resulting 200 structures, twenty lowest–energy structures were minimized with Amber 8 [20] and selected to represent the ChiAVD structure in solution. The coordinates of the final ensemble have been deposited to the RCSB Protein Data Bank (http://www.rcsb.org/pdb/) with the accession code 2mf6. Structure figures were created with UCSF Chimera [21].

^15^N longitudinal relaxation time (T_1_) and transverse relaxation time (T_2_) data were acquired with the following time points: 10, 60, 110, 330, 660, 900, 1100, 1500, 1800, 2600, 3500 ms (T_1_) and 10, 30, 50, 70, 90, 110 ms (T_2_). Duplicate spectra were acquired for estimation of uncertainties. Recycle delays were set to 3.1 s. R_1_ and R_2_ values were obtained by non–linear least–squares fitting of peak heights to a one–parameter exponential function using Curvefit (A. G. Palmer III, Columbia University). Uncertainties in the fitted parameters were obtained with Jackknife simulations (A. G. Palmer III, Columbia University). The {^1^H}–^15^N heteronuclear nuclear Overhauser enhancement (hetNOE) values were determined as the peak intensity ratio observed in NOE spectra acquired with and without ^1^H saturation. A recycle delay of 5.1 s was used in the hetNOE experiments. Proton saturation was applied for 5 s. An estimate of the error was obtained from the rms noise in the two spectra. Relaxation data were acquired at 58°C for the biotin–free form and at 40 and 58°C for the bound form.

Local correlation times were calculated with the program r2r1_tm (A. G. Palmer III, Columbia University) from trimmed R_2_/R_1_ data [22]. The global isotropic correlation time was calculated as the mean of these residue–specific values. The relative moments of the inertia tensor determined from the bound ChiAVD structure are 1.00∶0.79∶0.73. The molecular diffusion tensor was determined from a subset of residues in secondary structure regions and with no large–amplitude motions [22]. For the 800 MHz, 58°C, biotin–bound protein data this set included 51 out of 96 residues having relaxation data. The best fit to the experimental R_1_ and R_2_ values is obtained with an axially symmetric (oblate) tensor. The components of the tensor are D_⊥_ 0.13×10^−7^ and D_∥_ 0.12×10^−7^ s^−1^, θ–1.8°, φ 28.4°, resulting in a small degree of anisotropy (D_∥_/D_⊥_) of 0.90. The principal axes of the diffusion tensor are almost collinear with those of the inertia tensor, the maximum angle between the axes being ∼2.5°. Model–free analysis of the relaxation data at the two magnetic fields was performed with the program Tensor [23], with a version of the program allowing the number of residues to be up to 1000, kindly provided by M. Blackledge, Institut de Biologie Structurale (Grenoble, France).

H/D exchange experiments were carried out by first lyophilizing a sample in D_2_O and then dissolving it to H_2_O. Increase in cross peak intensity was followed by measuring ^1^H, ^15^N HSQC spectra at 58°C, 800 MHz. The first time point was at approximately 15 minutes after dissolution. The last time point for biotin–free ChiAVD sample was at 96 hours, and for the bound form weeks after dissolution. Peak intensities were fitted to a three–parameter equation of the form I(t) = I(∞)+I(0)*(1−exp(−*k*_ex_×t)). Residue specific protection factors [24], [25] were derived from the H/D exchange rates *k_ex_* using the spread sheet available from the Englander lab's website, http://hx2.med.upenn.edu/download.html

### RDC measurements

As ChiAVD is positively charged at the sample pH, we used bicelles, composed of 5% (w/V) DMPC/DHPC phospholipids at a molar ratio of 3∶1, as the liquid crystal medium. Due to the instability of this liquid crystal medium at elevated temperatures we measured ^1^H–^15^N RDCs at 40°C. As no deuterium labelling was utilized, we employed a modified version of the MQ–HNCO–TROSY experiment that has been successfully used for measuring ^1^H–^15^N RDCs in the 558–residue Filamin A 16–21 fragment [26].

RDCs were applied as constraints in all four subunits.

### Molecular dynamics simulations

The X–ray crystallographic structure of chimeric avidin [8] (PDB identifier 3MM0) was completed for the missing residues in L6,7 of chains E, F, G, and H (1–3 residues each) and L3,4 of chain G (Asn43) using Modeller 9v5 [27]. Biotin was placed into the binding pockets of ChiAVD with the help of wild–type avidin [4], PDB identifier 2AVI). Cocrystallized water molecules from within a 5 Å radius were included, and waters clashing with biotin were removed. Hydrogens were added using PDB2PQR 1.3.0. [28], [29]. GAFF parameters were assigned for the biotin using the antechamber module, and Amber_99SB parameters [30] were assigned for the protein in the tleap module in Amber 10 [20]. The tetrameric protein with or without biotin was placed in a 75 Å×86 Å×90 Å box filled with TIP3P water molecules. 11 and 15 Cl^−^ ions were added, resulting in a total of 52844 and 52973 atoms in the bound and ligand–free systems, respectively. Energy minimizations and molecular dynamics simulations were carried out in NAMD 2.6 [31]. Three 4000–step conjugate gradient minimizations were carried out for the ligand–bound complex: first with protein and ligand frozen, second with the ligand and Cα atoms frozen, and third without restraints. For the ligand–free system the second minimization was omitted. The systems were heated from 0 to 310 K in 31 ps and equilibrated for 2 ns in 310 K. The simulations were continued for 10 ns at three temperatures: 310, 333, and 523 K. The simulations were carried out in NPT conditions (1 atm) using the Berendsen thermostat and barostat. A 1–fs timestep was used in all simulations. The trajectory was superimposed by the Cα atoms using the RMSD Visualizer Tool plugin, and root mean square fluctuation (RMSF) was calculated using the “measure rmsf” command and a 1–ps step in VMD 1.9.1 [32].

### ChiAVD mutants G42A and G42F

Glycine 42 mutations to alanine and phenylalanine were done with conventional PCR mutagenesis. Protein expression was performed using pET101/D-TOPO vector (Invitrogen, Carlsbad, CA, USA) in BL21-AI *E. coli* (Invitrogen). Protein purification with 2-iminobiotin agarose (Affiland, Belgium) was performed as described in [16].

The dissociation rate constant (*k*_diss_) of fluorescently labelled ArcDiaTM BF560 (ArcDia, Turku, Finland) biotin was determined by fluorescence spectrometry essentially as described in [33]. The assay was performed at 50°C using a QuantaMasterTM Spectrofluorometer (Photon Technology International, Inc., Lawrenceville, NJ, USA) equipped with circulating water bath thermostat. The fluorescence probe was excited at 560 nm and emission was measured at 578 nm.

Determination of hydrodynamic radius was performed by dynamic light scattering (DLS) using Zetasizer Nano ZS (Malvern Instruments Ltd) in 50 mM NaH_2_PO_4_/Na_2_HPO_4_, 100 mM NaCl, pH 7 at 25°C. Six measurements were performed each consisting of 10 × 10 s measurement. Data was analysed using Zetasizer software v7.01 (Malvern Instruments Ltd) using “General purpose” model and volume distribution.

The transition midpoint (*T*_m_) of the ChiAVD forms was measured by differential scanning calorimetry (DSC) using VP-capillary DSC (GE Healthcare, MicroCal) in 50 mM NaH_2_PO_4_/Na_2_HPO_4_, 100 mM NaCl, pH 7. The scanning was carried out from 20°C to 140°C at a rate of 120°C/h, using a 5 s filter period and a low feedback mode. Measurements were done using 13.8 µM protein in the presence or absence of 36 µM D-biotin (Fluka prod. no. 14400). Data analysis was made using the Origin 7 software (GE Healthcare, MicroCal). The *T*_m_s were determined using a Non-2-state fitting model.

## Results and Discussion

### Structure of biotin–bound ChiAVD

We have solved the first solution structure of a member of the avidin–family of proteins. The structure of this 56 kDa protein was solved without resort to the laborious, yet relatively expensive method in which perdeuteration is combined with selective methyl protonation [34]. The deciding factor was the thermostability of the protein – a raise of the measurement temperature to 58°C reduces the protein's overall rotational correlational time (∼25 ns at room temperature) to half the value. Amide proton exchange rate with solvent significantly increases, however, along with temperature. To address this problem, a set of methyl proton detection experiments with high sensitivity and resolution [14] were employed in the assignment of methyl containing residues [16].

The homotetrameric symmetrical structure simplifies the NMR spectra of ChiAVD at the expense of losing potential NOE distance restraints at the very center of the protein structure (Arg114, Val115) where intra– and intersubunit correlations are indistinguishable. Intermolecular NOE cross peaks, here referring to those between ChiAVD subunits, were manually assigned from heteronuclear–edited NOE spectra. Identification of these peaks was based on an analysis of subunit interfaces of existing avidin crystal structures, mainly that of biotin–free ChiAVD [8]. To overcome the symmetry issue it would have been possible to create a tetrameric ChiAVD with differentially labelled subunits by using the methodology utilized earlier to produce monomeric avidin which tetramerizes upon addition of biotin [35]. Alternatively, dual chain avidin technology could have been applied [36].

Several NOE cross peaks from ChiAVD to biotin were observed in the NOE spectra. Despite numerous attempts with different concepts, we were unfortunately unable to obtain unambiguous chemical shift assignments for the bound biotin. We thus excluded these cross peaks from structure calculations. Additional restraints for structure calculation were obtained from chemical shifts, *J*–coupling constants, H/D exchange experiments, and ^1^ *D*_NH_ RDCs.

#### RDC measurements

**Figure 2:**
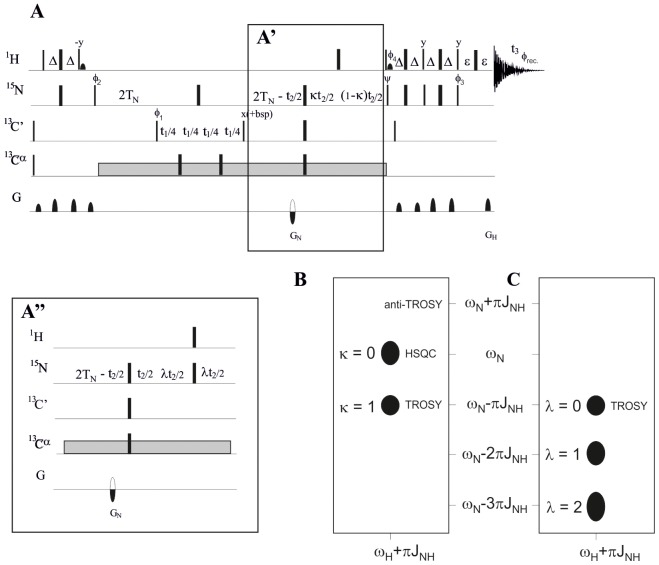
MQ-HNCO-TROSY+ experiment for the measurement of ^15^N-^1^H residual dipolar couplings in ^15^N, ^13^C, (^2^H) labeled proteins. (A) Narrow and wide black bars denote rf pulses with 90° and 180° flip angles, respectively. If not otherwise denoted, the pulses are applied with phase x. The ^1^H, ^15^N, ^13^C′, and ^13^Cα carrier positions are 4.7 (water), 118 (center of ^15^N spectral region), 175 ppm (center of ^13^C′ spectral region), and 57 ppm (center of ^13^Cα spectral region). 90° (180°) pulses for ^13^C′ are applied with a strength of Ω/√15 (Ω/√3), where Ω is the frequency difference between the centers of the ^13^C′ and the aliphatic ^13^Cα regions. The ^13^C carrier is placed in the middle of ^13^C′ region (175 ppm) and rectangular 180° pulses are applied off-resonance for ^13^Cα with phase modulation by Ω. Removal of ^13^C′–^13^Cα and ^15^N–^13^Cα coupling interactions during t_1_ and t_2_, respectively, can be accomplished using either the SEDUCE-1 decoupling sequence [37] or three 180° ^13^Cα rectangular pulses applied off-resonance with phase modulation by Ω. The delays used for coherence transfer are: Δ = 1/(4J_NH_); TN = 1/(4J_NC_′)  = 12.5–16.6 ms; ε =  duration of gradient + recovery delay. Inset (A′) shows implementation to select the anti-TROSY component, which is downscaled by a factor of κ (0<κ<1) with respect to the TROSY component (see panel B). The phase cycling used is: φ_1_ = x, −x; φ_2_ = x; φ_3_ = −x; φ_4_ = −x; ψ = −x; φ_rec._ = x, −x. Inset (A") shows pulse sequence implementation to select the anti-TROSY component which is scaled up by a factor of λ (λ>0) with respect to the TROSY component (see panel C). The phase cycling used is: φ_1_ = x, −x; φ_2_ = x; φ_3_ = −x; φ_4_ = −x; ψ = x; φ_rec._ = x, −x. Hence, for measuring ^1^J_NH_ (and ^1^(J+D)_NH_) couplings, the κ and λ values can be selected independently, for instance using κ = 0; λ = 1 yields two subspectra whose resonance frequencies differ by 2πJ_NH_ i.e. ^1^J_NH_ couplings can be obtained directly from the frequency separation. Quadrature detection in the indirect ^15^N (t_2_) dimension, the 90°(^15^N) with the phase ψ is inverted simultaneously with the gradient G_N_ to obtain echo/antiecho selection. The data processing is according to the sensitivity enhanced method [38]. The axial peaks are shifted to the edge of the spectrum by inverting φ_2_ together with φ_rec._ in every second t_2_ increment. Quadrature detection in the ^13^C′ dimension is obtained by States-TPPI protocol applied to φ_1_ [39].

**Figure 3:**
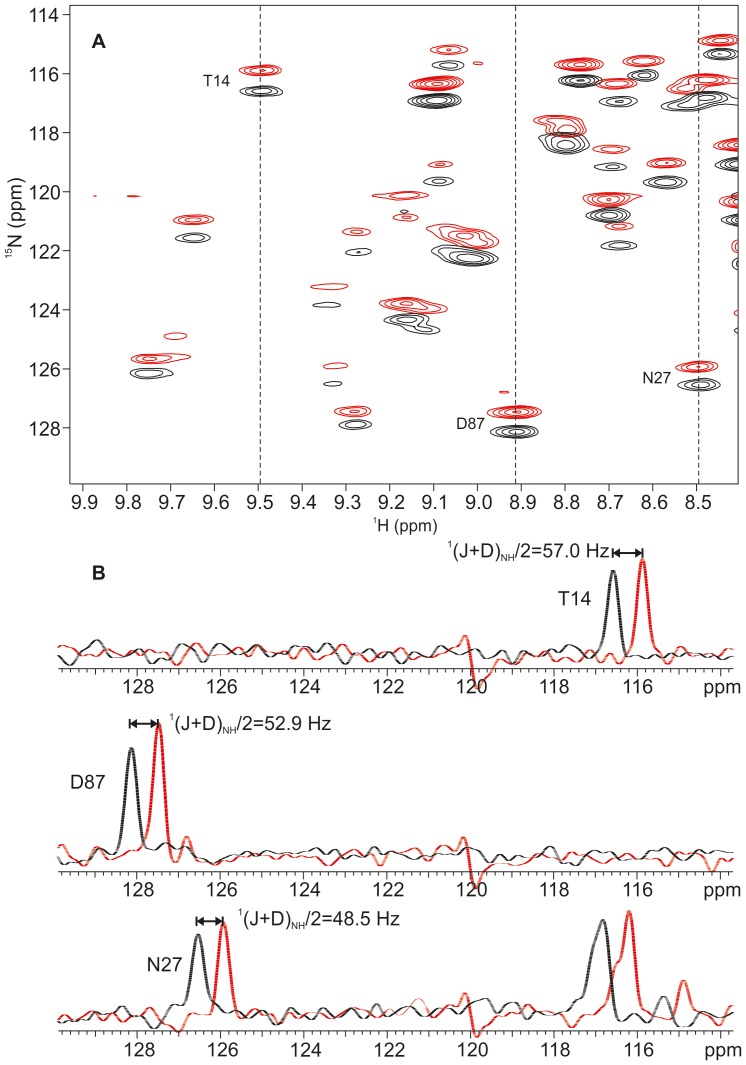
Excerpt from the MQ-HNCO-TROSY+ spectrum. The MQ-HNCO-TROSY+ spectrum of ^15^N, ^13^C labeled chimeric avidin was measured at 40°C on 800 MHz in a dilute liquid crystal medium composed of 3∶1 mixture of DMPC:DHPC phospholipids (bicelles). Panel (**A**) displays overlaid upfield (red contours) and downfield (black contours) components of ^15^N-{^1^H} doublets. Vertical dotted lines indicate the position of the corresponding ^15^N traces shown in panel (**B**) for T14, N27 and D87. As the scaling parameters were set to κ = 0.5 and λ = 0.5, the measured couplings are scaled down by the factor of 2. The measured apparent ^1^(J+D)_NH_/2 couplings are shown next to each splitting for T14, D87 and N27.

To measure ^1^ *D*_NH_ RDCs in ChiAVD, the newly modified MQ–HNCO–TROSY scheme (MQ–HNCO–TROSY+, Figure 2) was devised. The pulse scheme reduces losses associated to exchange broadening due to solvent exchange or *J* couplings. This experiment enables the determination of ^1^H–^15^N RDCs by measuring ^1^J_NH_ (in water) and ^1^(J+D)_NH_ (in liquid crystal medium) splittings in the ^15^N dimension between two anti–TROSY components whose relative position and effective linewidth, with respect to the TROSY component, can be fine–tuned with two parameters κ (0<κ<1) and λ (λ>0). In the case of ChiAVD, the ^1^(J+D)_NH_ splittings were obtained by recording two MQ–HNCO–TROSY+ spectra in an interleaved manner with κ = 0.5 and λ = 0.5 i.e. the apparent splitting measured in ^15^N dimension corresponds to ^1^(J+D)_NH_/2, hence doubling the random error. As highlighted in the figure, the upfield ^15^N–{^1^H} component generally exhibits a narrower linewidth with respect to the downfield ^15^N–{^1^H} component. The narrower linewidth and the increased S/N for the upfield component are due to a differential relaxation effect between the two experiments that select either the upfield or the downfield ^15^N–{^1^H} components. The former signal decays with a rate exp[–½(1–κ)(R_2A_–R_2T_)*t*_2_] and the latter with exp[–½λ(R_2A_+R_2T_)*t*_2_], where R_2A_ and R_2T_ are the transverse relaxation rates for the anti–TROSY and TROSY components. Therefore, in the MQ–HNCO–TROSY+ experiment, the linewidth for the upfield anti–TROSY component is always smaller than for the downfield component. This is especially pronounced in non–deuterated samples. The MQ-HNCO-TROSY+ spectrum of ^15^N, ^13^C labeled ChiAVD is shown in Figure 3.

In the case of ChiAVD, we were able to measure 93 RDCs. In structure calculation we used 58 RDC restraints omitting RDCs from the termini, more than half of RDCs originating from loops, as well as some RDC in secondary structure regions constantly giving large violations. Experimental RDCs, description of the alignment tensor and correlation plots are given in Figure S1 and Table S1. RDCs slightly improved the precision of the ensemble of ChiAVD structures, on average 0.06 Å for the backbone atoms of the ordered part of the sequence, residues 5–123. The RMSD to the biotin–free structure also marginally improved (0.03 Å for the same atoms).

**Figure 4:**
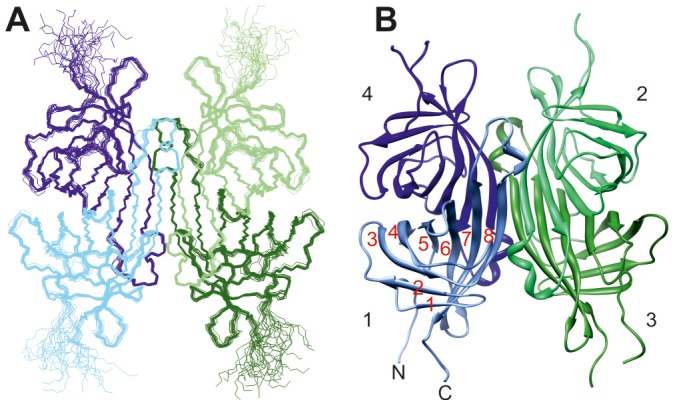
Solution NMR structure of biotin–bound ChiAVD. (**A**) Ensemble of fifteen structures of least restraint violations. (**B**) Lowest–energy structure represented as a ribbon diagram. In each monomer the protein is structured from residues Lys3 to Leu123. β strands in the mean structure span residues 8–12, 17–20, 28–34, 47–53, 63–69, 76–86, 90–100, and 113–122. Residues 106–111 coil to a 3_10_ helix.

An ensemble of biotin–bound ChiAVD structures with good precision was achieved (Figure 4 and Table 1). The backbone and heavy atom RMSDs to the mean monomer structure are 0.27 and 0.69 Å, respectively, and in the tetramer 0.32 and 0.71 Å. The RMSDs for the monomer and the tetramer are of the same order indicating that the relative orientation of the monomers and the monomer itself are structurally equally well defined.

**Table 1:** Structural Statistics of Biotin–bound ChiAVD.

Structural restraints per ChiAVD monomer						
Distance restraints						
		Intramolecular		2002		
		short–range |i–j| ≤1		917		
		medium–range 1<|i–j| <5		188		
		long–range |i–j| ≥5		897		
		hydrogen bond restraints		27		
		intermolecular, 1–2 interface		289		
		intermolecular, 1–3 interface		99		
Angle restraints						
		φ/ψ		68/67		
		χ		11		
N^H^–H^N^ RDC restraints				58		
Agreement with experimental data						
Average RMS deviations from restraints						
distance restraints (Å)					0.019±0.001	
dihedral restraints (°)					0.23±0.15	
RDC restraints (Hz)					0.55±0.02	
Average number of violations						
distance restraints >0.5 Å					0	
dihedral restraints >5°					0.4 (max. 8.4°)	
RDC restraints >1 Hz					3.7 (max. 2.3 Hz)	
Average RMS deviations from ideal covalent geometry						
bonds (Å)					0.011±0.002	
angles (°)					2.31±0.06	
Average RMS deviation from mean structure in ChiAVD tetramer (Å)^a^						
backbone atoms					0.23±0.04/0.32±0.05	
heavy atoms					0.59±0.05/0.71±0.04	
Ramachandran plot regions (%) in ChiAVD tetramer^b^						
most favored regions					83.4/87.4	
additionally allowed regions					13.6/11.5	
generously allowed ragions					2.1/0.8	
disallowed regions					0.9/0.3	

a Residues in β strands/Five residues excluded from the termini.

b All residues/Five residues excluded from the termini.

### Comparison of the structures of biotin–bound and free ChiAVD

**Figure 5:**
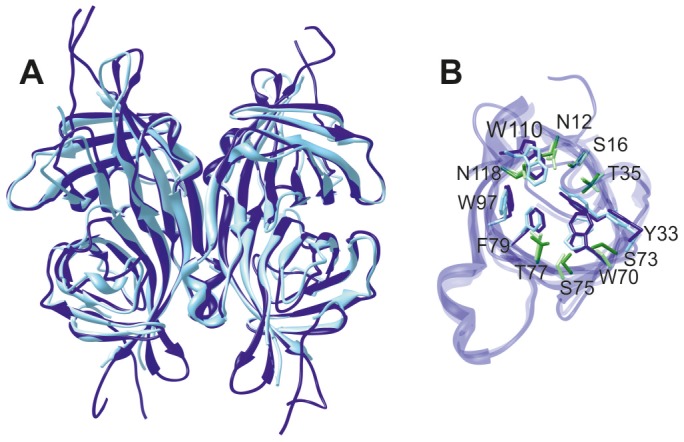
Overlay of the NMR structure of biotin–bound ChiAVD and the X–ray structure of biotin–free ChiAVD. (**A**) ChiAVD tetramer where the biotin–bound form is represented in dark blue and (**B**) biotin binding site residues. In (**B**) aromatic residues are shown in shades of blue (darker colors for the biotin–bound form) and polar residues in shades of green. The 1–2 interface brings Trp110 from the 1–2 related subunit to the binding site.

Despite the different experimental method and data collection conditions, namely the temperature, close structural similarity is evident for the two forms of ChiAVD. It is obvious that at 58°C the biotin-bound ChiAVD structure is still intact showing no indication of unfolding or dissociation into monomers. The β sheet regions of the biotin–bound ChiAVD solution structure superimpose well with those of the biotin–free form crystal structure [8] (Figure 5A). For the monomer the average backbone RMSD (N, Cα, C, O atoms in β strands, 232 atom pairs) is 0.73±0.04 Å and that for heavy atoms (N*, C*, O* atoms in β strands, 468 atom pairs), 1.24±0.04 Å. For the tetramer (960/1944 atom pairs) the RMSDs are slightly larger, 0.80±0.04 and 1.34±0.04 Å for the backbone and heavy atoms, respectively.

In loops the free *versus* bound RMSD values range from 0.81±0.10 Å for the four–residue loop connecting β strands 1 and 2 (L1,2, residues 13–16 of the tetramer) to 1.68±0.28 Å found for L4,5 (residues 54–62). The higher RMSDs in loops are the result of increased mobility as compared to the structured parts (see below the ^15^N relaxation analysis). Notably, this is true also for L3,4 (residues 35–46) in which the backbone atom RMSD to the free form is 1.29±0.13 Å. In the biotin–free form residues Pro41–Gly42 of this loop fold to a helix–like conformation (φ/ψ angles on average −64°/−9° and −69°/−22°) whereas in the biotin–bound form these residues are found in multiple conformations. None of the conformations precludes the formation of the stabilising intramonomeric salt bridge between side chains of Asp39 and Arg114 from β8 observed in the structures of biotin–free ChiAVD and AVR4 (Asp39–Arg112, [8], [9]). This salt bridge is present in half of the structures of the ensemble.

Although NOEs to biotin were excluded from the structure calculations, residues in the biotin binding site are well defined (Figure 5B). Some of the side chains of polar residues have, however, mutually different orientations. Besides the lack of biotin resonance assignments, here also the fact that no attempts were made to assign the side chain hydroxyl and amide group protons contributes to the differences observed.

### Biotin–free ChiAVD in solution

The biotin–free ChiAVD sample deteriorates substantially faster than that of the bound form at the high temperature needed for sufficient NMR experiment sensitivity. Cross peaks become wider and their shape gets distorted although remaining at same positions with no additional peaks appearing over time. We suspect that over time in the prevalent solution conditions the protein molecules transiently interact to form higher molecular weight states. Assignment of the backbone resonances was however successfully conducted at 70°C [16]. Methyl group chemical shifts of free ChiAVD are listed in Table S2.

The structural similarity of the bound and free forms is evident from chemical shift comparison. The Δδ Cα, Cβ, N, H^N^ (free–bound) persuasively show that the chemical environments within monomers differ uniquely at residues located in the biotin binding site (see Figure S2 for Δδ Cβ). The largest shift differences, up to 3–5 ppm, are observed in L3,4 for residues Val37 and Ala38. The ^1^H, ^13^C chemical shifts of methyl groups located at the interfaces also match. A comparison of the ^1^H, ^13^C HSQC spectra of free (at 80°C) and bound (58°C) ChiAVD reveals that the methyl–containing residues at the 1–4 dimer interface exhibit comparable side chain chemical shifts (Δδ(^13^C) <0.27 ppm, Δδ(^1^H) <0.04 ppm) in the two forms. No significant methyl chemical shift changes are observed for Met96, Thr113, or Val115 at the 1–2 and 1–3 interfaces either. The N^ε1^–H^ε1^ pair of Trp110 bridging monomers 1 and 2 shifts notably. This is, however, caused by direct interaction with biotin.

Because of the different acquisition temperatures, the ^1^H line widths in the ^1^H, ^13^C HSQC spectra of free and bound ChiAVD cannot be directly compared. However, the measured line widths in both spectra can be divided in to three categories depending on their magnitude. We observe that each methyl resides in the same line width category in both protein states. We deduce that in the two forms the methyl groups at the interfaces have similar dynamical characteristics arising from similar chemical environments.

In all, the chemical shift and line width data indicate a close tertiary and quaternary structure similarity between the free and the bound form. It is thus justified to use the structure of the bound form in solution at 58°C in the analysis of the ^15^N relaxation data of both forms. When extracting diffusion tensor parameters from the relaxation data this structure also gave lower χ^2^ target function values as compared to the crystal structure of the free form. Details of the diffusion tensor parameters and their derivation are given in Figure S3.

### Model–free analysis

R_1_, R_2_ and heteronuclear Overhauser (hetNOE) ^15^N relaxation data were recorded on 600 and 800 MHz spectrometers at 58°C for free and bound ChiAVD. For the bound form also relaxation data at 40°C on 800 MHz were recorded. These data as well as the average values are presented in Figure S4 and Table S3. The relaxation data were interpreted using the Model–free approach [40], [41] and are presented for the 800 MHz data. Similar results were obtained from the analysis of the 600 MHz data.

Assuming isotropic diffusion, the overall rotational correlation times (τ_c_) are 13.0±0.4 ns for the biotin–bound ChiAVD, and 13.2±0.4 for the free form at 58°C. At 40°C, a τ_c_ of 18.0±0.6 ns is found for the bound form. By assuming a linear correlation between the ratio of solvent viscosity and temperature (η/T) and τ_c_, at 25°C ChiAVD has an overall rotational correlation time of 25.4 ns. It is interesting to note, that this *t_c_* differs markedly from that estimated by the empirical formula, τ_c_ = 0.5998×MW+0.1674, giving 33.8 ns. The Stokes-Einstein relation for the reorientation of a hard sphere gives an estimate of 21.0 ns for τ_c_ assuming a hydration radius of 3.2 Å. The observed rigidity of ChiAVD (see below) might lower the rotational correlation time towards the value predicted by the latter relation. This is consistent with the notably low τ_c_ s found for the highly rigid β-lactamases TEM-1 [42] and PSE-4 [43].

**Figure 6:**
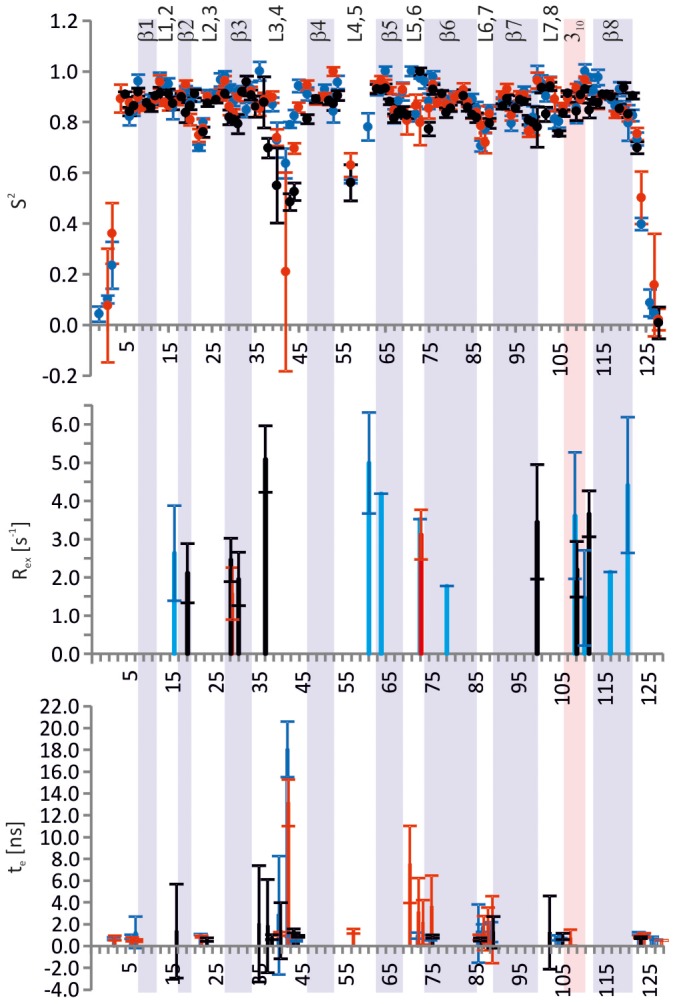
Model–free parameters of ChiAVD. S^2^, R_ex_ and τ_e_ (>0.5 ns) of ChiAVD in biotin–free form at 58°C are shown in black and those of the bound form at 40°C in blue and 58°C in red. The secondary structure of the biotin–bound structure at 58°C is also shown.

The Model–free parameters S^2^, τ_e_ and R_ex_ are presented in Figure 6. The squared order parameter, S^2^, provides information on the amplitude of the N–H bond vector motion. It varies between 0 and 1, with 1 corresponding to completely restricted bond vector motions and 0 to completely unrestricted motions. τ_e_ gives the effective time scale of the bond vector motion, typically ranging from 0 to 100 ps but in loops and termini up to a few ns. R_ex_ reports on the presence of µs-ms time scale exchange.

#### Comparison of free and bound ChiAVD

On average the two forms are dynamically very similar but subtle differences are observed in loop regions. Average S^2^, excluding the termini, are 0.86±0.03 for the bound and 0.85±0.02 for the free form. Secondary structure regions have an average S^2^ of 0.89±0.02 in the bound and 0.87±0.02 in the free form. Altogether both forms are remarkably stable taking into account the high measurement temperature.

Corresponding data are available for 66 residues. Large reduction (ΔS^2^>0.1) in the amplitude of motion upon biotin binding is observed for residues Thr19 (β2), Gly31 (β3), Ala38, Asn40 and Ile44 (L3,4), Gln53 (β4) and Arg100 (β7). The most impressive change is observed in L3,4 with ΔS^2^ values of 0.13–0.19. Mobility in L3,4 is however still present in the biotin−bound form as Asn40 and Ile44 have S^2^ values of ∼0.7 and Gly42 as low as 0.2. Two residues, Ser73 and Phe120, show significant increase in mobility upon biotin binding.

Two residues exhibit modest values of R_ex_ (1.6≤R_ex_≤3.1 s^−1^) in the bound form, and seven residues in the free form, with slightly larger values (2.0≤R_ex_≤5.1 s^−1^). No correlation of R_ex_ with H/D exchange data (see below) is detected. Instead, the presence of R_ex_ for Val37, Arg100, Asp109 and Ala112 in the free form could possibly be associated with missing stabilizing side–chain interactions with biotin present in the bound form. The first of these is located in the lid–making loop whereas the three others, located in the protrusion of the β–barrel, are in contact with the 1–2–related monomer via Trp110.

In both formsτ_e_ is relaxation active for several residues in loops and in a few loop–flanking residues. Largest contributions of τ_e_ to relaxation are observed for residues in L3,4 and L5,6, with the bound form values outweighing those of the free form.

#### Comparison of bound ChiAVD at two different temperatures

The rigidity of the protein is almost completely retained when increasing the temperature from 40 to 58°C. The average S^2^ of 0.86±0.03 observed at 58°C for all residues excluding the flexible residues at the termini has only slightly decreased from the 0.88±0.03 observed at 40°C. If only residues in secondary structures are considered the same average S^2^ value is found for the two temperatures. Interestingly, a per residue analysis shows that it is the biotin–binding region that becomes more mobile when temperature is raised. Largest decrease in S^2^ are observed for residues Gly15, Ala36, Gly42, Ile44, Thr45, Ser73 and Arg114, all at the biotin–binding end of the β–barrel. Three of these are located in the lid–making loop L3,4. At the lower temperature the number of residues with a R_ex_ contribution is larger. Unexpectedly, the majority of the residues exhibiting slow exchange are located in secondary structure regions.

The Model–free parameters derived from the relaxation data imply that ChiAVD is a remarkably rigid protein. A search of entries including order parameters deposited in the BioMagResBank (http://www.bmrb.wisc.edu/) reveals that in the thirty data sets with an experimental temperature below 34°C the calculated average S^2^ (omitting possible flexible terminal residues) ranges from 0.71 to 0.92. Only a few deposited data sets with an experimental temperature above 40°C are available. In these the average S^2^ are 0.90 at 45°C for Trp repressor (BMRB entry 17041), 0.86 at 50°C for CtCBM11 (18389), 0.82–0.84 at 47–73°C for calmodulin–peptide complex (4970), and 0.81 at 44°C for azurin (6243). From the literature we find, in addition, average S^2^ of 0.54 (50°C) for the B1 domain of Streptococcal protein G [44], 0.82 (45°C) for cardiac troponin C [45], 0.81–0.76 at 15–47°C for ubiquitin [46], and 0.88 (β strands only, 45°C) for OspA [47]. Considering that for most proteins studied to date the temperature dependency of S^2^ is negative, ChiAVD with an average S^2^ of 0.88 (40°C) and 0.86 (58°C) ranks among the most rigid proteins studied. The current understanding that among factors potentially increasing chemical and thermal stability is the reduction of conformational flexibility [48] is nicely in line with the fact that ChiAVD is extremely stable towards harsh conditions. It is, however important to note that the ^15^N–^1^H vector motions represent only a subset of the backbone dynamics, and acquisition of motional data of the ^13^C′–^13^C^α^ vector would result in a more comprehensive perception of the overall backbone motions [49].

### Conformational entropy

The contribution of conformational entropy changes to binding free energy can be derived from the S^2^ values [50], [51]. For the 66 residues considered the net loss in conformational entropy is −122.9 J×mol^−1^K^−1^. This figure includes only the fast ps–ns time–scale motion of amide bond vectors of a subset of residues of the protein. It is however close to the experimentally determined ΔS of −115.7 J×mol^−1^K^−1^ found for the structurally related protein AVR4/5(C122S) [7] meaning that this type of motion makes a significant contribution to the entropic term of the Gibbs free energy of binding biotin to ChiAVD.

### H/D exchange

**Figure 7:**
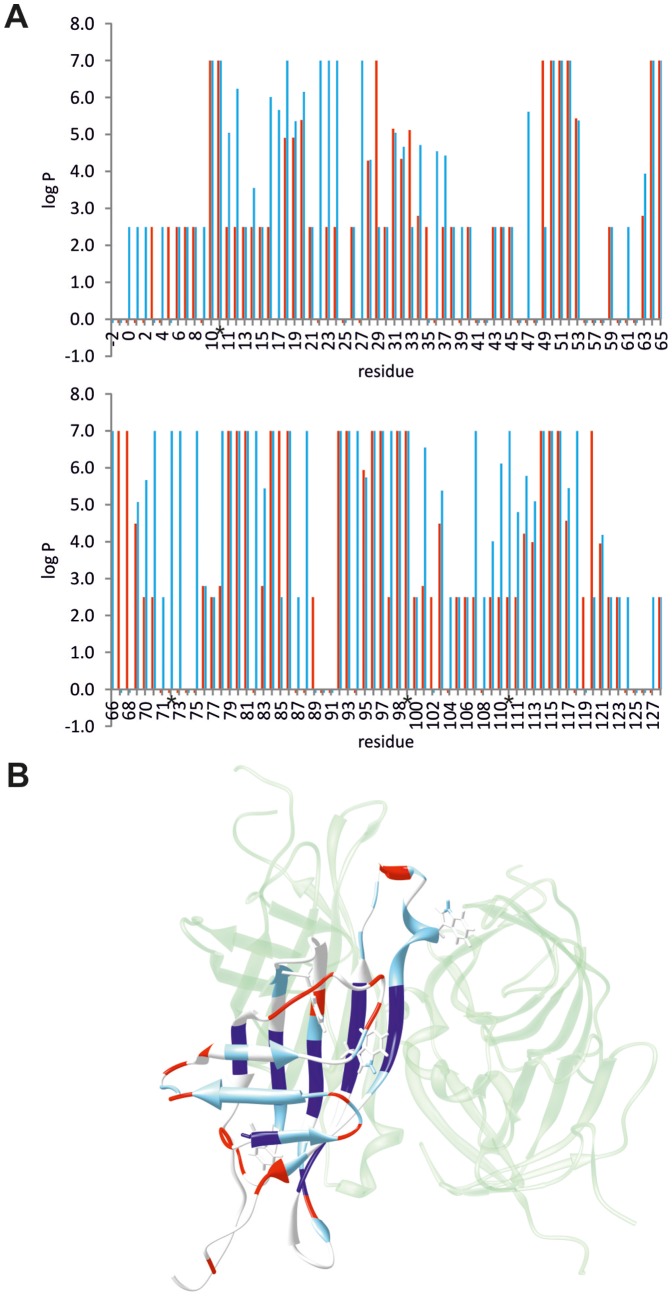
Per residue protection factors of biotin–free and bound ChiAVD. (**A**) The protection factors of the free form are shown with red bars. Residues exhibiting fast exchange have been assigned the value 2.5, residues in the second fastest group (see text) the value 2.8, and residues with slow exchange the value 7.0. Residues with no data available have been assigned the value −0.1. Trp side chain data are marked with asterisks. (**B**) Differences in protection factors shown in the structure of ChiAVD. In dark blue are shown residues with enhanced protection to exchange in both forms and in light blue residues for which the free form is more prone to exchange. Residues shown in red are susceptible to fast exchange in both forms. Residues coloured in white have no comparable data.

H/D exchange studies were performed at 58°C for the free and biotin–bound ChiAVD. Data (partly qualitative) were obtained for 89 (free) and 104 (bound) residues (see Figure 7 and Figure S5 showing the curve fitting to the data). Twenty–five (free) and thirty–seven (bound) backbone amide hydrogens as well as Trp10 (free) and Trp10, 70, 97, and 110 (bound) side chain H^ε1^ hydrogens are very efficiently buried and/or hydrogen bonded. Their ^1^H, ^15^N HSQC cross peak remain unperturbed for days after solvent exchange. Most of these are located at subunit interfaces: residues in β4–β7 at the 1–4 interface, residue 114 and residues 115–116 in β8 at the 1–2 and 1–3 interfaces, respectively. Trp70, 97 and 110 are essential in providing hydrophobic interactions to biotin. Trp10 is located at the opposite end of the β–barrel and forms, in both free and bound ChiAVD, a hydrogen–bond with its H^ε1^ to Leu6 carbonyl oxygen.

An estimate for the lower limit of the highest rate constant was calculated from peak intensities in the first spectrum after solvent addition [52]. Residues which had exchanged before the first time point have a rate constant of >2.9×10^−3^ s^−1^ (represented with a protection factor *P*, expressed as log *P*, of 2.5 in Figure 7). Forty and thirty–nine residues belong to this group in the free and bound ChiAVD, respectively. For residues which had exchanged before the second time point the approximate rate constant is 1.7×10^−3^<*k*_ex_<2.9×10^−3^ s^−1^ (log *P* 2.8). Six (free) and one (bound) residues belong to this group. Backbone amide protons of fifteen (free) and twenty–five (bound) residues exchanged within the experimental time. On average, the rates for free ChiAVD are 0.28×10^−3^ s^−1^ faster than for the bound form.

It is evident from these data that the bound form is considerably more stable in terms of H^N^ protection arising from hydrogen bonding and/or burial. The free and bound ChiAVD differ in H^N^ protection at the side facing solvent (residues in β1–β3) as well as in L7,8 and the following 3_10_ helix (Figure 7B). Similar results have been obtained for streptavidin by H/D exchange and mass spectrometric studies [53]. In both proteins β1–β3 at the solvent–exposed face of the structures, as well as the 3_10_ helix at the 1–2 interface show a reduction in exchange upon biotin binding. At the 1–4 interface a larger number of amide hydrogens are labile in the biotin–free form of streptavidin as compared to that of ChiAVD: In addition to residues in β5–β6 and β8 protected in both proteins, in ChiAVD solvent protection covers also residues in β7. H/D exchange and infrared spectroscopic studies with avidin [54] also indicated a reduction in the proportion of exchangeable hydrogens and reduction in the fast exchange kinetic constant upon biotin binding. ChiAVD has a biotin binding affinity similar to that of AVR4 [7], which is higher than that observed for streptavidin [55]. This mutual difference might partially be accounted for by the differences observed in hydrogen protection. In ChiAVD the more extensive hydrogen bond network in the free form would imply a smaller loss in entropy upon biotin binding which would have a favourable effect on the thermodynamics of the reaction and thus increase affinity. A further entropic benefit for ChiAVD might result from L3,4 retaining at least partially its mobility (again a smaller loss in entropy) as opposed to streptavidin in which a reduction of exchange is observed for the entire loop.

### MD analysis

**Figure 8:**
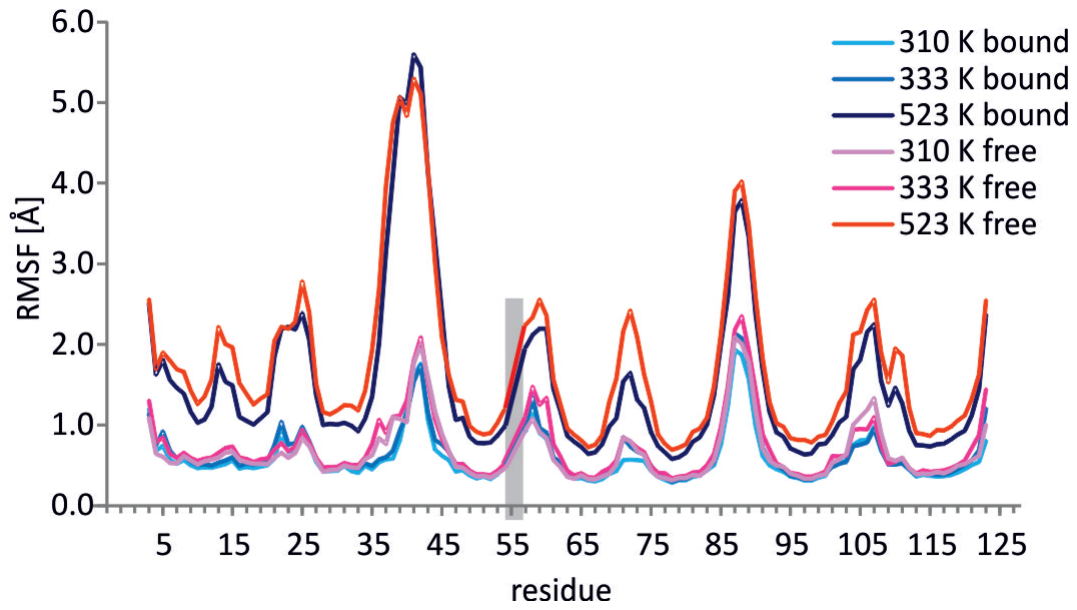
MD simulations of ChiAVD. RMSF at three temperatures is shown, in the absence and presence of biotin. The area shaded in grey marks the gap in the amino acid sequence numbering.

Molecular dynamics simulations were carried out at three temperatures for 10 ns. Movement of the protein chain is visualized by plotting the root mean square fluctuation over time (RMSF; Figure 8). A clear correlation between secondary structure and RMSF is observed: all eight β strands are found to show much lower fluctuation as compared to the loops connecting them. L3,4 and L6,7 are the most mobile. Biotin stabilizes most loops in all the temperatures tested, but the stabilizing effect is rather modest. Using an average of five residues to calculate the ΔRMSF, the highest degree of stabilization is observed in sequence stretches Thr35–Asp39 for 310 K (ΔRMSF: −32%) and 333 K (ΔRMSF: −32%) and Trp70–Phe74 for 523 K (ΔRMSF: −31%). RMSF is found to correlate with the temperature used. Movement of Ala22 in L2,3 increases in the presence of biotin.

The results of the molecular dynamics simulation agree remarkably well with the order parameter data, with the exception of L6,7. The simulation nicely exposes the small differences observed in the experimental data between the free and the bound protein form, namely the larger number of mobile residues in L3,4 of the free form and the loop's overall higher amplitude of motion. Also the lower stability of the 3_10_ helix in the free form is evident. However, the order parameters show no indication of high mobility for L6,7. In fact the simulation data indicates higher mobility for twelve residues, encompassing half of the β strands flanking the three–residue L6,7.

### ChiAVD mutants G42A and G42F

Apart from the termini, three regions stand out from the average motional regime in ChiAVD: L3,4, L4,5 and L5,6. According to the S^2^ and τ_e_ values residue Gly42 in L3,4 is the most mobile residue. In quest of further stability, we mutated Gly42 to alanine and phenylalanine. The Gly→Ala mutation represents the simplest attempt to create rigidity with a bulkier side chain whereas the objective of the Gly→Phe mutation was to create a stabilizing π–π aromatic interaction with Phe72 in the structurally opposite L5,6. (Strept)avidins have extensively been modified by mutagenesis [56], but to our knowledge the outcome of mutating Gly42 has not yet been described. Intriguingly, Chivers et al. [57] applied point mutations S52G and R53D to streptavidin (streptavidin Ser52 equals to ChiAVD Gly42 in sequence) and obtained streptavidin with both decreased dissociation and association rates, but also with increased thermal stability.

The mutants expressed efficiently (data not shown), folded properly to tetramers and had physical properties very similar to ChiAVD. A hydrodynamic radius of 3.04±0.75 nm was measured by DLS for ChiAVD–G42A, 3.09±0.70 nm for ChiAVD–G42F, and 3.18±0.69 nm for ChiAVD. The main peak consisted of 99.7–100% of the volume–adjusted intensity. The obtained hydrodynamic size corresponds to globular protein with molecular weights of 45.4 kDa (ChiAVD–G42A), 47.2 kDa (ChiAVD–G42F) and 50.4 kDa (ChiAVD), which is very close to the theoretical size of the tetramer.

Mutation of Gly42 had no notable influence on the thermostability. However, we observed a slightly lower biotin dissociation rate for the mutants. At 50°C ChiAVD–G42A showed a slightly lower fluorescently labelled biotin dissociation rate than ChiAVD–G42F or ChiAVD: (4.64±0.33)×10^−5^ s^−1^ as compared to (5.80±0.40) or (6.39±0.88) ×10^−5^ s^−1^, indicating that both of the mutations were slightly beneficial for the binding of the conjugated biotin. The *T_m_* values obtained from DSC experiments were 110.5±0.2/129.6±1.0°C for biotin–free/bound ChiAVD, 110.1±0.0/128.8±0.0°C for ChiAVD–G42A, and 109.9±0.0/128.2±0.1°C for ChiAVD–G42F. We hypothesise that the introduced bulkier sidechains do not form the pursued, thermostability enhancing, stable interactions to their structural neighbors. Instead, the occasional interactions hinder the loop dynamics. A resulting smaller gain in entropy upon biotin release for the mutants would reduce the dissociation rate.

## Conclusions

We have shown that at 58°C biotin-bound ChiAVD maintains its compact, well-defined structure. With an average order parameter S^2^ of 0.86, it is one of the most rigid proteins studied to date. Rigidity plays a significant role in promoting stability. The extent of the subunit interfaces, reflected in the high number of very slowly exchanging amide hydrogens also contributes to the stability. The biotin-free forms of the avidin family of proteins are less stable than the bound forms. For ChiAVD the difference in stability can be (at least partially) ascribed to the smaller extent of H-bonding/burial of amide hydrogens and higher nano-picosecond flexibility (S^2^) at the biotin-binding end of the β-barrel as compared to the bound form.

## Supporting Information

Figure S1 **Correlation plots of ChiAVD experimental and calculated RDCs.** (**A**) ChiAVD experimental RDCs *vs* RDCs calculated from the biotin-bound ChiAVD NMR structure. (**B**) ChiAVD experimental RDCs *vs* RDCs calculated from the biotin-free ChiAVD X-ray structure, PDB id 3MM0.
(TIF)

Figure S2 **Chemical shift differences between free and biotin-bound chimeric avidin.** (**A**) The bar graph represents the Cβ chemical shift difference between free and bound forms of ChiAVD. In (**B**) locations of residues showing a large (≥0.7 ppm) Cβ chemical shift difference are shown in the structure of chimeric avidin. The Cβ atoms are shown as spheres. Biotin was included in the structure by superimposition with the structure of AVR4 (PDB id 1Y52).
(TIF)

Figure S3 **Relative orientations and parameters of diffusion tensors calculated from the 800 MHz R_1_, R_2_ data.** Diffusion tensor calculations were done with the program TENSOR2. The fitted parameters D_‖_ and D_⊥_ are shown here as D_xx_, D_yy_, D_zz_ for direct comparison with the asymmetric tensor: for the oblate tensor (1^st^ minimun) D_‖_ = D_xx_ and D_⊥_ = D_zz_ = D_yy_, for the prolate tensor (2^nd^ minimum) D_‖_ = D_zz_ and D_⊥_ = D_xx_ = D_yy_. The angles α, β and γ describe the orientatation of the diffusion tensor in the structure frame. For axially symmetric tensor, the polar angles φ and θ are given.
(TIF)

Figure S4 **^15^N R_1_, R_2_, and hetNOE data of biotin–bound and free ChiAVD.** The 800 MHz data are represented with red (58°C) and blue (40°C) dots, and those of 600 MHz (58°C) with black dots. Trp N^ε1^ data at 800 MHz, 58°C are shown with green dots. Secondary structure regions are highlighted with β strands in blue and 3_10_ helices in red. The depicted secondary structure of the free form is that present in the crystal structure [1]. Average errors are 0.04 (800 MHz, 58°C), 0.03 (800 MHz, 40°C), and 0.03 (600 MHz, 58°C) s^−1^ for R_1_, 0.40/0.70/0.31 s^−1^ for R_2_ and 0.04/0.04/0.06 for hetNOE in the bound form and 0.03/0.03 (800/600 MHz, R_1_), 0.31/0.48 (R_2_) and 0.05/0.04 (hetNOE) for the free form. The amino acid sequence has a gap (highlighted in grey): His54 is followed by Lys57. The first three residues Gln(−3), Thr(−2) and Val(−1) do not belong to the original mature protein form, but result from the signal peptide construction used in the bacterial expression.
(TIF)

Figure S5 **Representative curves of H/D exchange experiments.** Free ChiAVD is represented with empty circles and the bound form with filled circles.
(TIF)

Table S1 **ChiAVD experimental RDCs measured at 40°C, 800 MHz.**
(TIF)

Table S2 **Methyl group chemical shifts of free ChiAVD at 80°C.**
(TIF)

Table S3 **Average R_1_, R_2_ and hetNOE values and standard deviations for biotin–bound and free ChiAVD.**
(TIF)

## References

[1] Diamandis EP, Christopoulos TK (1991) The biotin-(strept)avidin system: Principles and applications in biotechnology. Clin Chem 37: 625–636. 2032315

[2] Wilchek M, Bayer EA (1999) Editorial. Biomol Eng 16: 1–4. 10796978 10.1016/s1050-3862(99)00032-7

[3] Laitinen OH, Nordlund HR, Hytönen VP, Kulomaa MS (2007) Brave new (strept)avidins in biotechnology. Trends Biotechnol 25: 269–277. 17433846 10.1016/j.tibtech.2007.04.001

[4] Livnah O, Bayer EA, Wilchek M, Sussman JL (1993) Three-dimensional structures of avidin and the avidin-biotin complex. Proc Natl Acad Sci U S A 90: 5076–5080. 8506353 10.1073/pnas.90.11.5076 PMC46657

[5] Sano T, Cantor CR (1995) Intersubunit contacts made by tryptophan 120 with biotin are essential for both strong biotin binding and biotin-induced tighter subunit association of streptavidin. Proc Natl Acad Sci U S A 92: 3180–3184. 7724536 10.1073/pnas.92.8.3180 PMC42129

[6] Gonzalez M, Argarana CE, Fidelio GD (1999) Extremely high thermal stability of streptavidin and avidin upon biotin binding. Biomol Eng 16: 67–72. 10796986 10.1016/s1050-3862(99)00041-8

[7] Hytönen VP, Määttä JA, Nyholm TK, Livnah O, Eisenberg-Domovich Y, et al (2005) Design and construction of highly stable, protease-resistant chimeric avidins. J Biol Chem 280: 10228–10233. 15649900 10.1074/jbc.M414196200

[8] Määttä JA, Eisenberg-Domovich Y, Nordlund HR, Hayouka R, Kulomaa MS, et al (2011) Chimeric avidin shows stability against harsh chemical conditions–biochemical analysis and 3D structure. Biotechnol Bioeng 108: 481–490. 20939005 10.1002/bit.22962

[9] Eisenberg-Domovich Y, Hytönen VP, Wilchek M, Bayer EA, Kulomaa MS, et al (2005) High-resolution crystal structure of an avidin-related protein: Insight into high-affinity biotin binding and protein stability. Acta Crystallogr D Biol Crystallogr 61: 528–538. 15858262 10.1107/S0907444905003914

[10] Hytönen VP, Nyholm TK, Pentikäinen OT, Vaarno J, Porkka EJ, et al (2004) Chicken avidin-related protein 4/5 shows superior thermal stability when compared with avidin while retaining high affinity to biotin. J Biol Chem 279: 9337–9343. 14660583 10.1074/jbc.M310989200

[11] Laitinen OH, Hytönen VP, Ahlroth MK, Pentikäinen OT, Gallagher C, et al (2002) Chicken avidin-related proteins show altered biotin-binding and physico-chemical properties as compared with avidin. Biochem J 363: 609–617. 11964162 10.1042/0264-6021:3630609 PMC1222514

[12] Heikkinen JJ, Kivimäki L, Määttä JA, Mäkelä I, Hakalahti L, et al (2011) Versatile bio-ink for covalent immobilization of chimeric avidin on sol-gel substrates. Colloids Surf B Biointerfaces 87: 409–414. 21705202 10.1016/j.colsurfb.2011.05.052

[13] Heikkinen JJ, Riihimäki TA, Määttä JA, Suomela SE, Kantomaa J, et al (2011) Covalent biofunctionalization of cellulose acetate with thermostable chimeric avidin. ACS Appl Mater Interfaces 3: 2240–2245. 21612241 10.1021/am200272u

[14] Würtz P, Hellman M, Tossavainen H, Permi P (2006) Towards unambiguous assignment of methyl-containing residues by double and triple sensitivity-enhanced HCCmHm-TOCSY experiments. J Biomol NMR 36: 13–26. 10.1007/s10858-006-9056-3 16964533

[15] Torchia DA (2011) Dynamics of biomolecules from picoseconds to seconds at atomic resolution. J Magn Reson 212: 1–10. 21840740 10.1016/j.jmr.2011.07.010

[16] Tossavainen H, Helppolainen SH, Määttä JA, Pihlajamaa T, Hytönen VP, et al (2013) Resonance assignments of the 56 kDa chimeric avidin in the biotin-bound and free forms. Biomol NMR Assign 7: 35–38. 22392339 10.1007/s12104-012-9371-4

[17] López-Méndez B, Güntert P (2006) Automated protein structure determination from NMR spectra. J Am Chem Soc 128: 13112–13122. 17017791 10.1021/ja061136l

[18] Cornilescu G, Delaglio F, Bax A (1999) Protein backbone angle restraints from searching a database for chemical shift and sequence homology. J Biomol NMR 13: 289–302. 10212987 10.1023/a:1008392405740

[19] Schwieters CD, Kuszewski JJ, Tjandra N, Clore GM (2003) The xplor-NIH NMR molecular structure determination package. J Magn Reson 160: 65–73. 12565051 10.1016/s1090-7807(02)00014-9

[20] Case DA, Cheatham ITE, Darden T, Gohlke H, Luo R, et al (2005) The amber biomolecular simulation programs. J Computat Chem 26: 1668–1688. 10.1002/jcc.20290 PMC1989667 16200636

[21] Pettersen EF, Goddard TD, Huang CC, Couch GS, Greenblatt DM, et al (2004) UCSF chimera–a visualization system for exploratory research and analysis. J Comput Chem 25: 1605–1612. 15264254 10.1002/jcc.20084

[22] Tjandra N, Wingfield P, Stahl S, Bax A (1996) Anisotropic rotational diffusion of perdeuterated HIV protease from 15N NMR relaxation measurements at two magnetic fields. J Biomol NMR 8: 273–284. 8953218 10.1007/BF00410326

[23] Dosset P, Hus JC, Blackledge M, Marion D (2000) Efficient analysis of macromolecular rotational diffusion from heteronuclear relaxation data. J Biomol NMR 16: 23–28. 10718609 10.1023/a:1008305808620

[24] Bai Y, Milne JS, Mayne L, Englander SW (1993) Primary structure effects on peptide group hydrogen exchange. Proteins 17: 75–86. 8234246 10.1002/prot.340170110 PMC3438223

[25] Connelly GP, Bai Y, Jeng M-F, Englander SW (1993) Isotope effects in peptide group hydrogen exchange. Proteins 17: 87–92. 8234247 10.1002/prot.340170111

[26] Mäntylahti S, Koskela O, Jiang P, Permi P (2010) MQ-HNCO-TROSY for the measurement of scalar and residual dipolar couplings in larger proteins: Application to a 557-residue IgFLNa16-21. J Biomol NMR 47: 183–194. 20454834 10.1007/s10858-010-9422-z

[27] Sali A, Blundell TL (1993) Comparative protein modelling by satisfaction of spatial restraints. J Mol Biol 234: 779–815. 8254673 10.1006/jmbi.1993.1626

[28] Dolinsky TJ, Nielsen JE, McCammon JA, Baker NA (2004) PDB2PQR: An automated pipeline for the setup of poisson-boltzmann electrostatics calculations. Nucleic Acids Res 32: W665–W667. 15215472 10.1093/nar/gkh381 PMC441519

[29] Dolinsky TJ, Czodrowski P, Li H, Nielsen JE, Jensen JH, et al (2007) PDB2PQR: Expanding and upgrading automated preparation of biomolecular structures for molecular simulations. Nucleic Acids Res 35: W522–W525. 17488841 10.1093/nar/gkm276 PMC1933214

[30] Hornak V, Abel R, Okur A, Strockbine B, Roitberg A, et al (2006) Comparison of multiple amber force fields and development of improved protein backbone parameters. Proteins 65: 712–725. 16981200 10.1002/prot.21123 PMC4805110

[31] Phillips JC, Braun R, Wang W, Gumbart J, Tajkhorshid E, et al (2005) Scalable molecular dynamics with NAMD. J Comput Chem 26: 1781–1802. 16222654 10.1002/jcc.20289 PMC2486339

[32] Humphrey W, Dalke A, Schulten K (1996) VMD: Visual molecular dynamics. J Mol Graph 14: : 33–8, 27–8. 10.1016/0263-7855(96)00018-5 8744570

[33] Hytönen VP, Laitinen OH, Airenne TT, Kidron H, Meltola NJ, et al (2004) Efficient production of active chicken avidin using a bacterial signal peptide in escherichia coli. Biochem J 384: 385–390. 15324300 10.1042/BJ20041114 PMC1134122

[34] Goto NK, Gardner KH, Mueller GA, Willis RC, Kay LE (1999) A robust and cost-effective method for the production of val, leu, ile (delta 1) methyl-protonated 15N-, 13C-, 2H-labeled proteins. J Biomol NMR 13: 369–374. 10383198 10.1023/a:1008393201236

[35] Laitinen OH, Marttila AT, Airenne KJ, Kulik T, Livnah O, et al (2001) Biotin induces tetramerization of a recombinant monomeric avidin. A model for protein-protein interactions. J Biol Chem 276: 8219–8224. 11076945 10.1074/jbc.M007930200

[36] Hytönen VP, Nordlund HR, Hörhä J, Nyholm TK, Hyre DE, et al (2005) Dual-affinity avidin molecules. Proteins 61: 597–607. 16175628 10.1002/prot.20604

[37] McCoy MA, Mueller L (1992) Selective shaped pulse decoupling in NMR: homonuclear [carbon-13]carbonyl decoupling. J Am Chem Soc 114: 2108–2112.

[38] Kay LE, Keifer P, Saarinen T (1992) Pure absorption gradient enhanced heteronuclear single quantum correlation spectroscopy with improved sensitivity. J Am Chem Soc 114: 10663–10665.

[39] Marion D, Ikura M, Tschudin R, Bax A (1989) Rapid recording of 2D NMR spectra without phase cycling. Application to the study of hydrogen exchange in proteins. J Magn Reson 85: 393–399.

[40] Lipari G, Szabo A (1982) Model-free approach to the interpretation of nuclear magnetic resonance relaxation in macromolecules. 1. theory and range of validity. J Am Chem Soc 104: 4546–4559.

[41] Lipari G, Szabo A (1982) Model-free approach to the interpretation of nuclear magnetic resonance relaxation in macromolecules. 2. analysis of experimental results. J Am Chem Soc 104: 4559–4570.

[42] Savard P-Y, Gagné SM (2006) Backbone dynamics of TEM-1 determined by NMR: Evidence for a highly ordered protein. Biochemistry 45: 11414–11424. 16981701 10.1021/bi060414q

[43] Morin S, Gagné SM (2009) NMR dynamics of PSE-4 β-lactamase: An interplay of ps-ns order and µs-ms motions in the active site. Biophys J 96: 4681–4691. 19486690 10.1016/j.bpj.2009.02.068 PMC2711454

[44] Seewald MJ, Pichumani K, Stowell C, Tibbals BV, Regan L, et al (2000) The role of backbone conformational heat capacity in protein stability: Temperature dependent dynamics of the B1 domain of streptococcal protein G. Protein Sci 9: 1177–1193. 10892810 10.1110/ps.9.6.1177 PMC2144655

[45] Spyracopoulos L, Lavigne P, Crump MP, Gagné SM, Kay CM, et al (2001) Temperature dependence of dynamics and thermodynamics of the regulatory domain of human cardiac troponin C. Biochemistry. 40: 12541–12551. 10.1021/bi010903k 11601978

[46] Chang SL, Tjandra N (2005) Temperature dependence of protein backbone motion from carbonyl 13C and amide 15N NMR relaxation. J Magn Reson 174: 43–53. 15809171 10.1016/j.jmr.2005.01.008

[47] Pawley NH, Koide S, Nicholson LK (2002) Backbone dynamics and thermodynamics of borrelia outer surface protein A. J Mol Biol. 324: 991–1002. 10.1016/s0022-2836(02)01146-4 12470954

[48] Vieille C, Zeikus GJ (2001) Hyperthermophilic enzymes: Sources, uses, and molecular mechanisms for thermostability. Microbiol Mol Biol Rev 65: 1–43. 11238984 10.1128/MMBR.65.1.1-43.2001 PMC99017

[49] Wang T, Cai S, Zuiderweg ER (2003) Temperature dependence of anisotropic protein backbone dynamics. J Am Chem Soc 125: 8639–8643. 12848571 10.1021/ja034077+

[50] Akke M, Bruschweiler R, Palmer AG 3rd (1993) NMR order parameters and free energy: An analytical approach and its application to cooperative calcium(2+) binding by calbindin D9k. J Am Chem Soc 115: 9832–9833.

[51] Yang D, Kay LE (1996) Contributions to conformational entropy arising from bond vector fluctuations measured from NMR-derived order parameters: Application to protein folding. J Mol Biol 263: 369–382. 8913313 10.1006/jmbi.1996.0581

[52] Skelton NJ, Kördel J, Akke M, Chazin WJ (1992) Nuclear magnetic resonance studies of the internal dynamics in apo, (Cd2+)1 and (Ca2+)2 calbindin D9k. the rates of amide proton exchange with solvent. J Mol Biol 227: 1100–1117. 1331470 10.1016/0022-2836(92)90524-n

[53] Williams DH, Zhou M, Stephens E (2006) Ligand binding energy and enzyme efficiency from reductions in protein dynamics. J Mol Biol 355: 760–767. 16325850 10.1016/j.jmb.2005.11.015

[54] Celej MS, Montich GG, Fidelio GD (2004) Conformational flexibility of avidin: The influence of biotin binding. Biochem Biophys Res Commun 325: 922–927. 15541378 10.1016/j.bbrc.2004.10.118

[55] Hytönen VP, Määttä JA, Kidron H, Halling KK, Hörhä J, et al (2005) Avidin related protein 2 shows unique structural and functional features among the avidin protein family. BMC Biotechnol 5: 28. 16212654 10.1186/1472-6750-5-28 PMC1282572

[56] Laitinen OH, Hytönen VP, Nordlund HR, Kulomaa MS (2006) Genetically engineered avidins and streptavidins. Cell Mol Life Sci 63: 2992–3017. 17086379 10.1007/s00018-006-6288-z PMC11136427

[57] Chivers CE, Crozat E, Chu C, Moy VT, Sherratt D, et al (2010) A streptavidin variant with slower biotin dissociation and increased mechanostability. Nat Methods 7: 391–393. 20383133 10.1038/nmeth.1450 PMC2862113

