# Supplementary figures and images for: Chimeric Avidin – NMR Structure and Dynamics of a 56 kDa Homotetrameric Thermostable Protein
Source: PLoS One. 2014 Jun 24;9(6):e100564. doi: 10.1371/journal.pone.0100564 (PMC4069078; doi:10.1371/journal.pone.0100564)

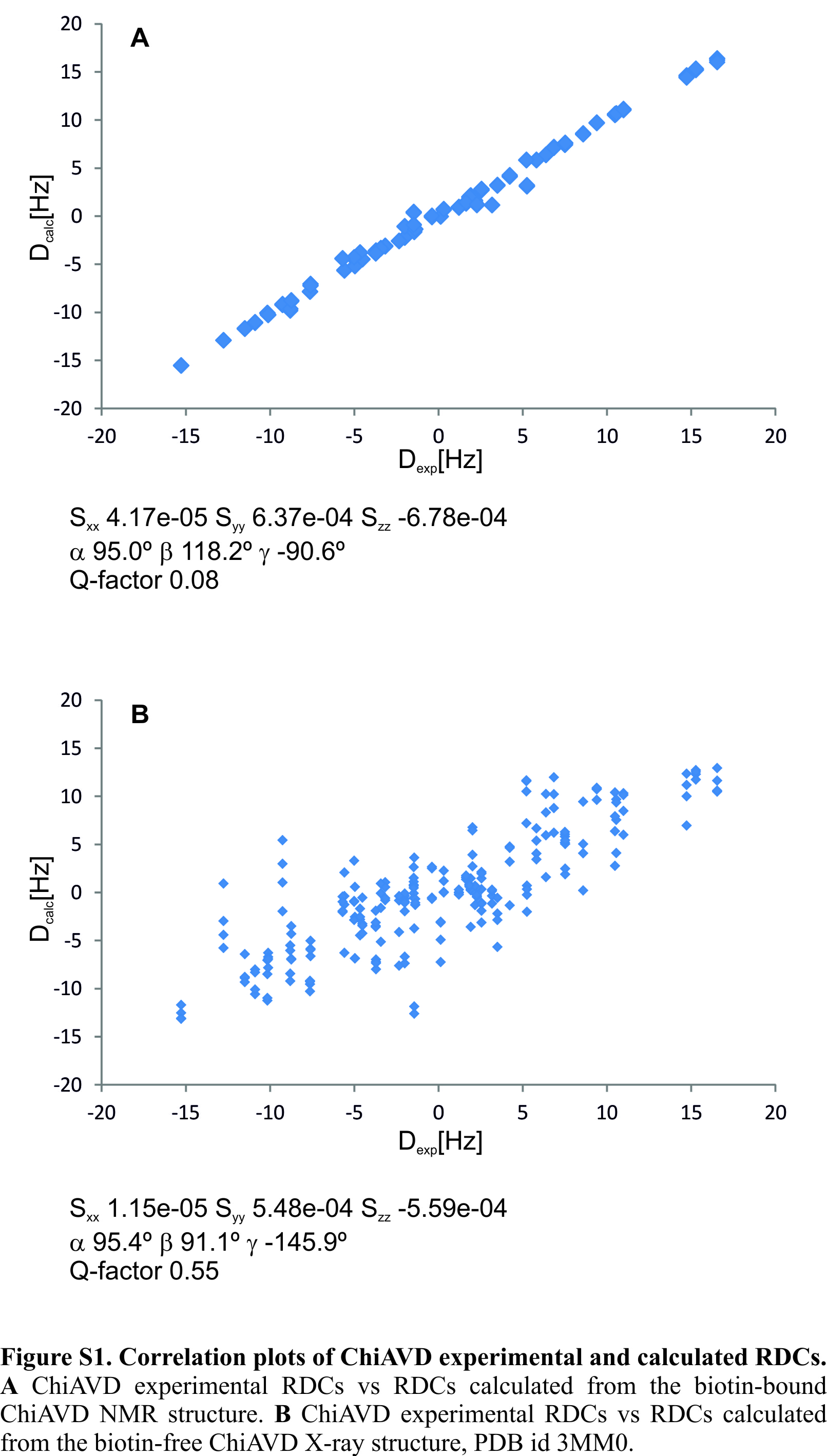

Supplement: Figure S1 — Correlation plots of ChiAVD experimental and calculated RDCs. (A) ChiAVD experimental RDCs vs RDCs calculated from the biotin-bound ChiAVD NMR structure. (B) ChiAVD experimental RDCs vs RDCs calculated from the biotin-free ChiAVD X-ray structure, PDB id 3MM0. (TIF) [file pone.0100564.s001.tif]

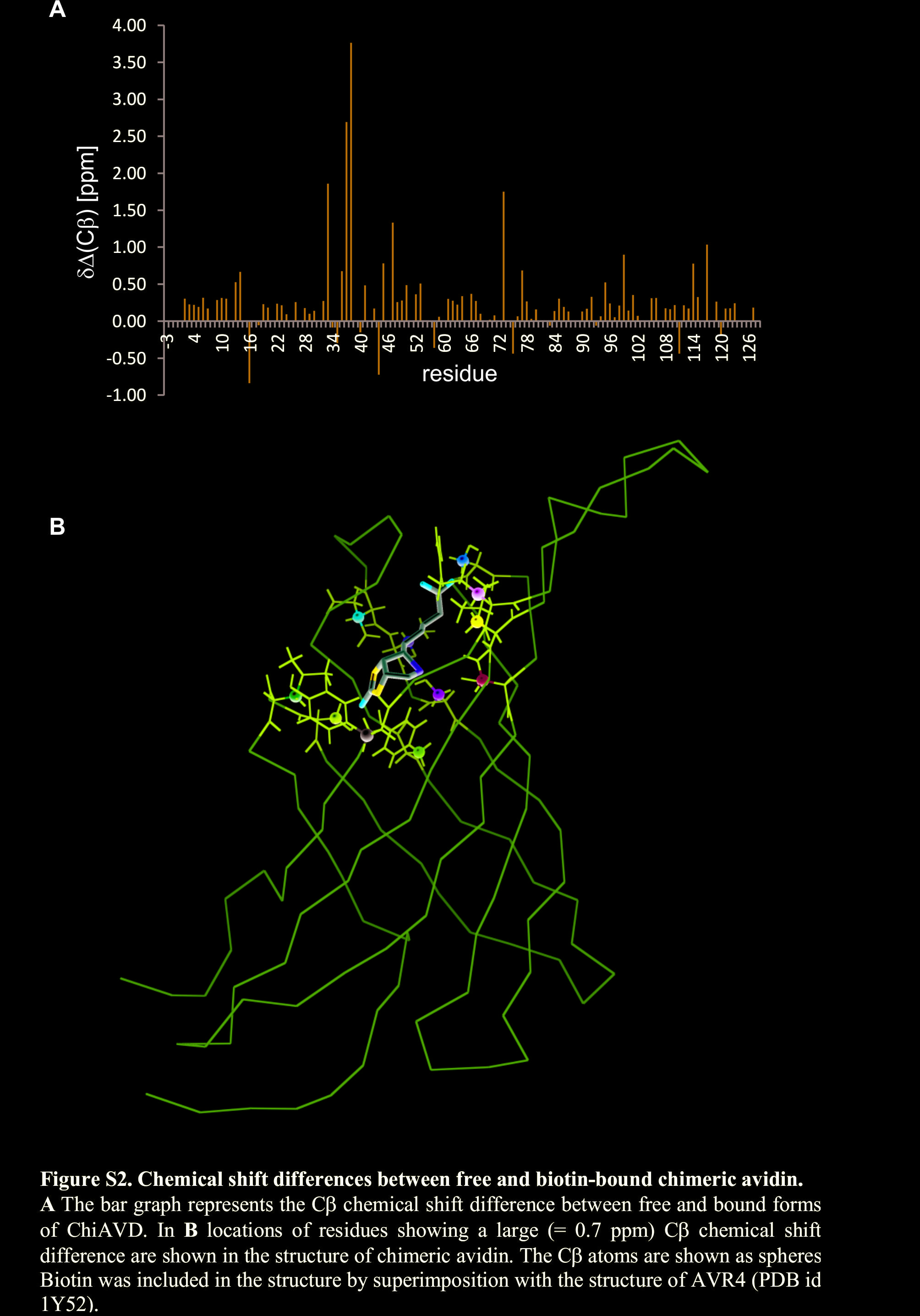

Supplement: Figure S2 — Chemical shift differences between free and biotin-bound chimeric avidin. (A) The bar graph represents the Cβ chemical shift difference between free and bound forms of ChiAVD. In (B) locations of residues showing a large (≥0.7 ppm) Cβ chemical shift difference are shown in the structure of chimeric avidin. The Cβ atoms are shown as spheres. Biotin was included in the structure by superimposition with the structure of AVR4 (PDB id 1Y52). (TIF) [file pone.0100564.s002.tif]

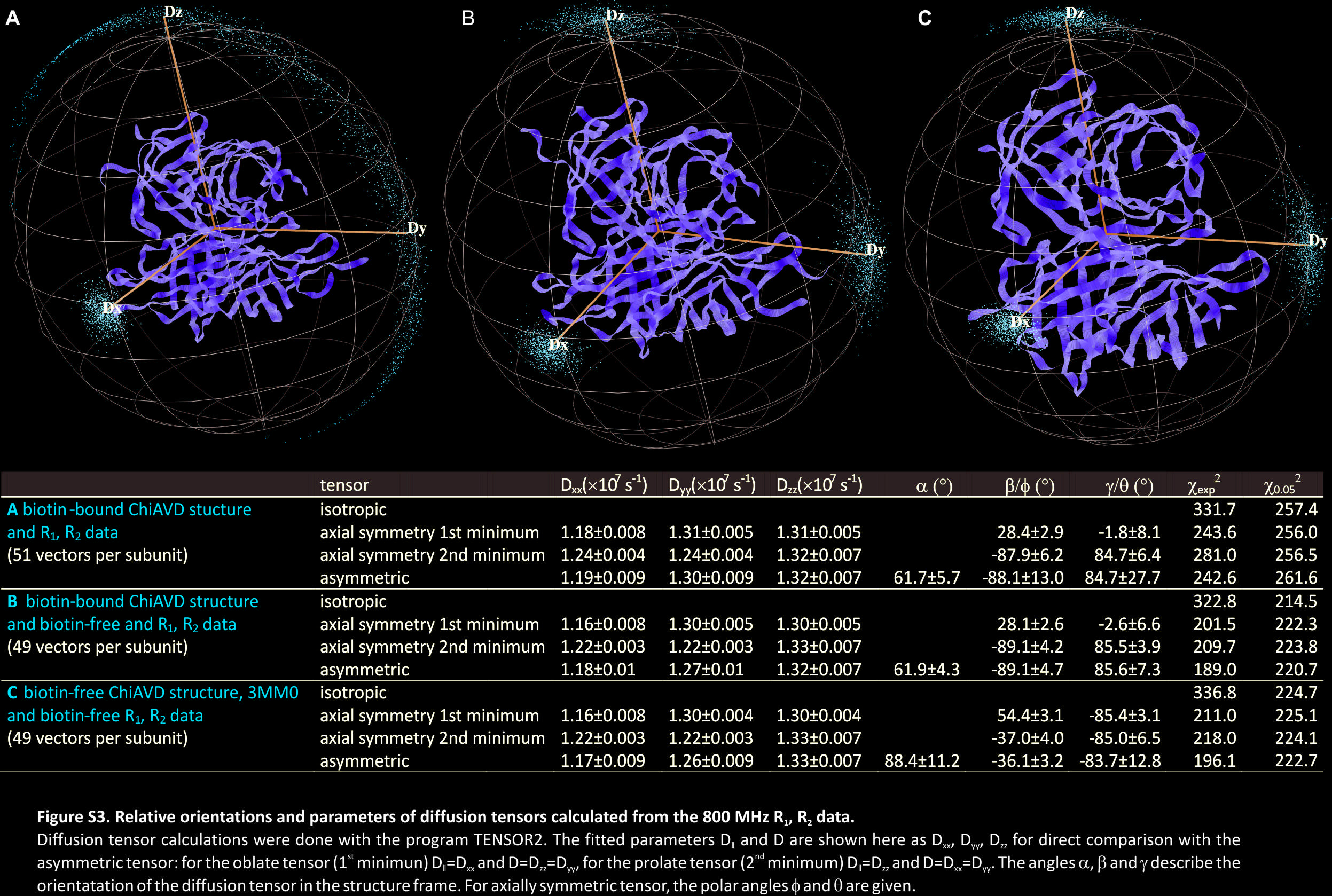

Supplement: Figure S3 — Relative orientations and parameters of diffusion tensors calculated from the 800 MHz R1, R2 data. Diffusion tensor calculations were done with the program TENSOR2. The fitted parameters D‖ and D⊥ are shown here as Dxx, Dyy, Dzz for direct comparison with the asymmetric tensor: for the oblate tensor (1st minimun) D‖ = Dxx and D⊥ = Dzz = Dyy, for the prolate tensor (2nd minimum) D‖ = Dzz and D⊥ = Dxx = Dyy. The angles α, β and γ describe the orientatation of the diffusion tensor in the structure frame. For axially symmetric tensor, the polar angles φ and θ are given. (TIF) [file pone.0100564.s003.tif]

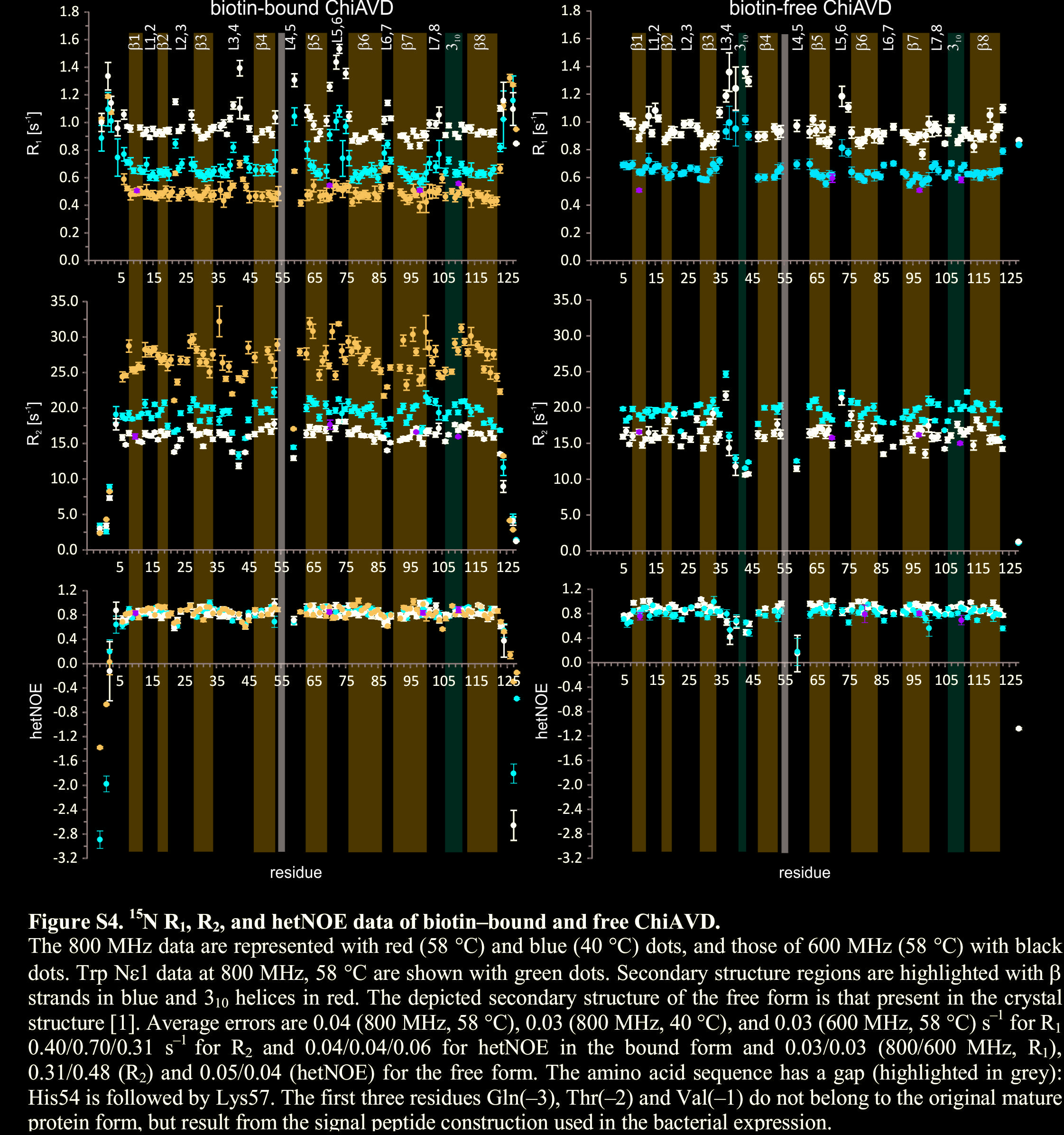

Supplement: Figure S4 — 15N R1, R2, and hetNOE data of biotin–bound and free ChiAVD. The 800 MHz data are represented with red (58°C) and blue (40°C) dots, and those of 600 MHz (58°C) with black dots. Trp Nε1 data at 800 MHz, 58°C are shown with green dots. Secondary structure regions are highlighted with β strands in blue and 310 helices in red. The depicted secondary structure of the free form is that present in the crystal structure [1]. Average errors are 0.04 (800 MHz, 58°C), 0.03 (800 MHz, 40°C), and 0.03 (600 MHz, 58°C) s−1 for R1, 0.40/0.70/0.31 s−1 for R2 and 0.04/0.04/0.06 for hetNOE in the bound form and 0.03/0.03 (800/600 MHz, R1), 0.31/0.48 (R2) and 0.05/0.04 (hetNOE) for the free form. The amino acid sequence has a gap (highlighted in grey): His54 is followed by Lys57. The first three residues Gln(−3), Thr(−2) and Val(−1) do not belong to the original mature protein form, but result from the signal peptide construction used in the bacterial expression. (TIF) [file pone.0100564.s004.tif]

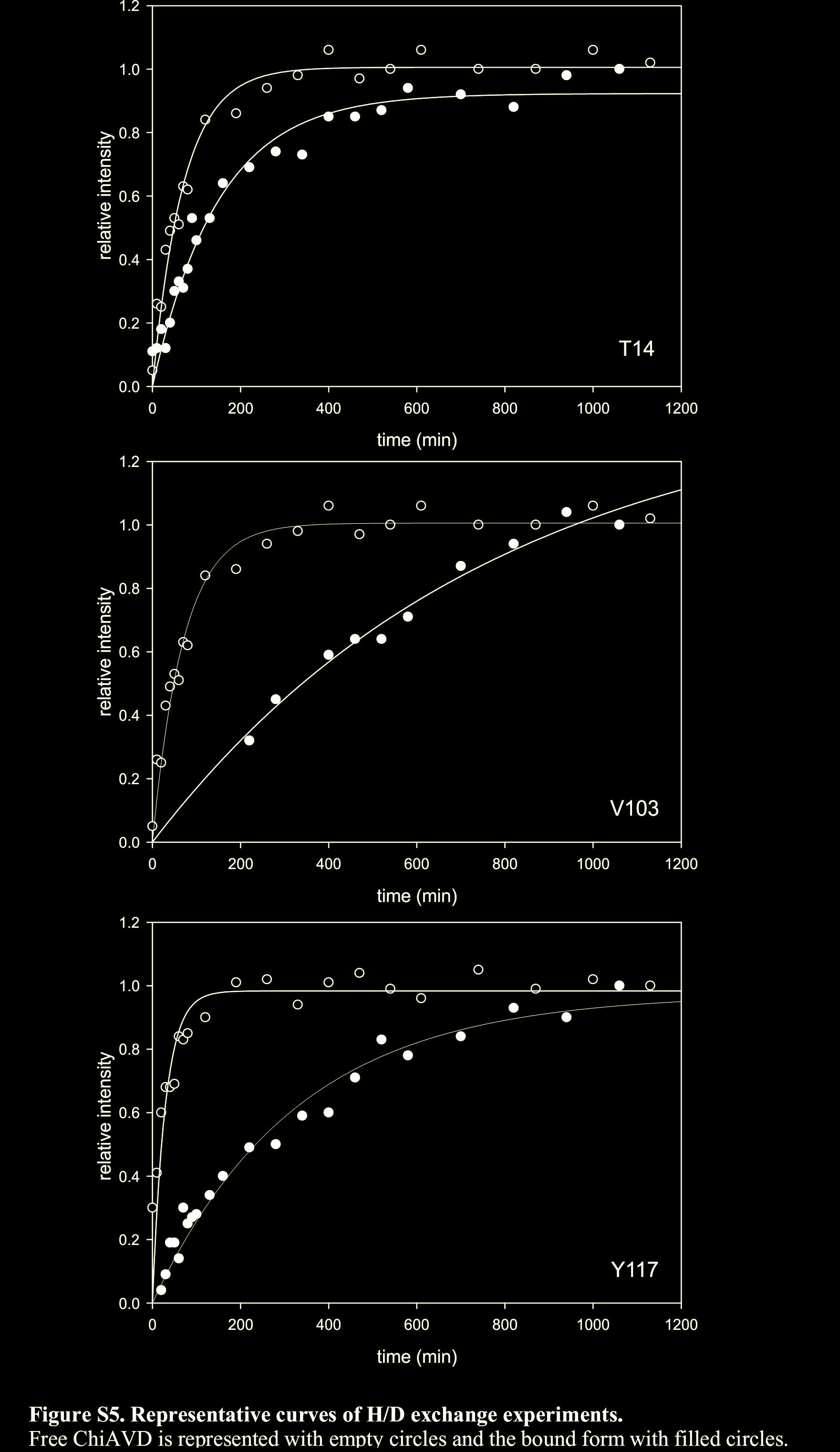

Supplement: Figure S5 — Representative curves of H/D exchange experiments. Free ChiAVD is represented with empty circles and the bound form with filled circles. (TIF) [file pone.0100564.s005.tif]

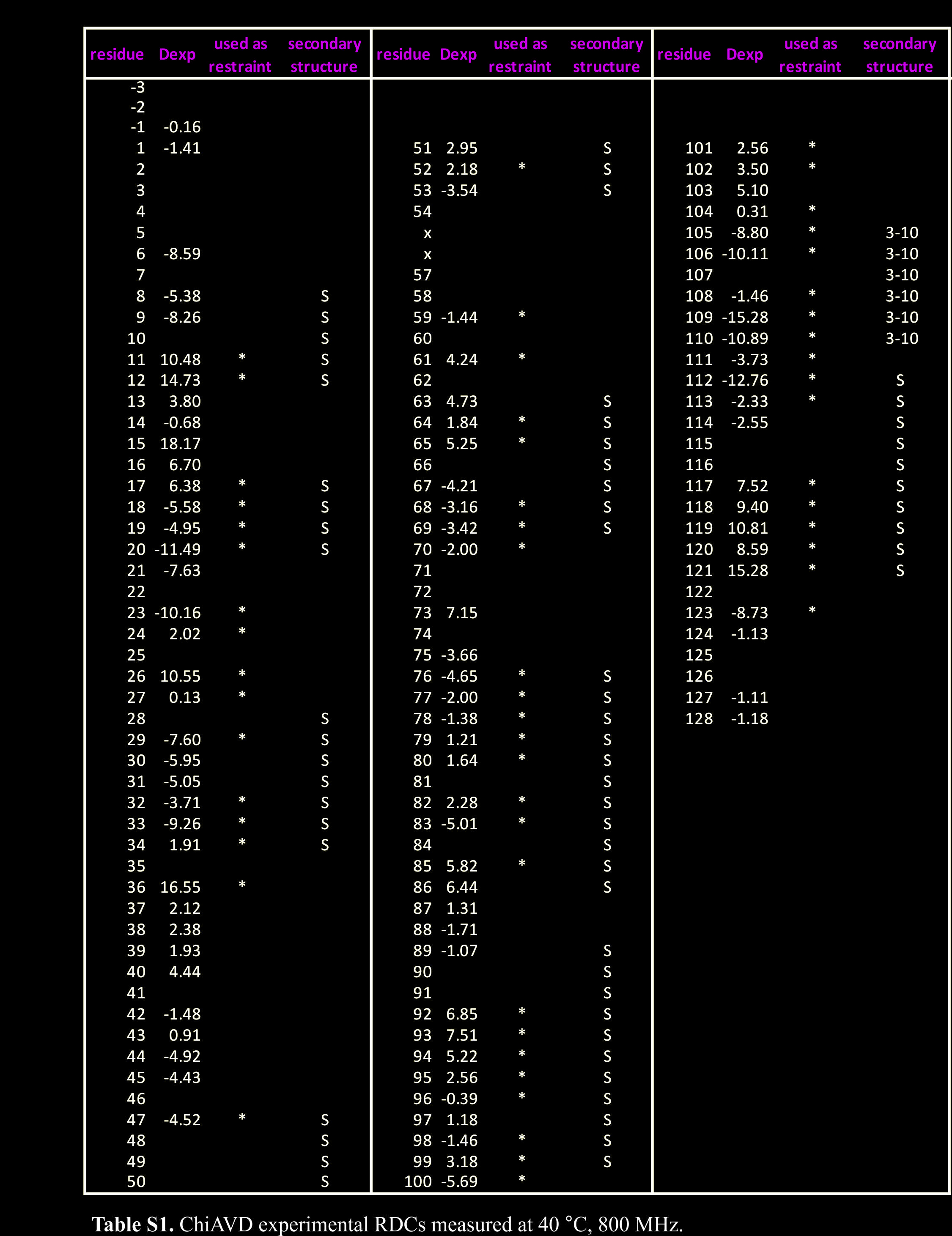

Supplement: Table S1 — ChiAVD experimental RDCs measured at 40°C, 800 MHz. (TIF) [file pone.0100564.s006.tif]

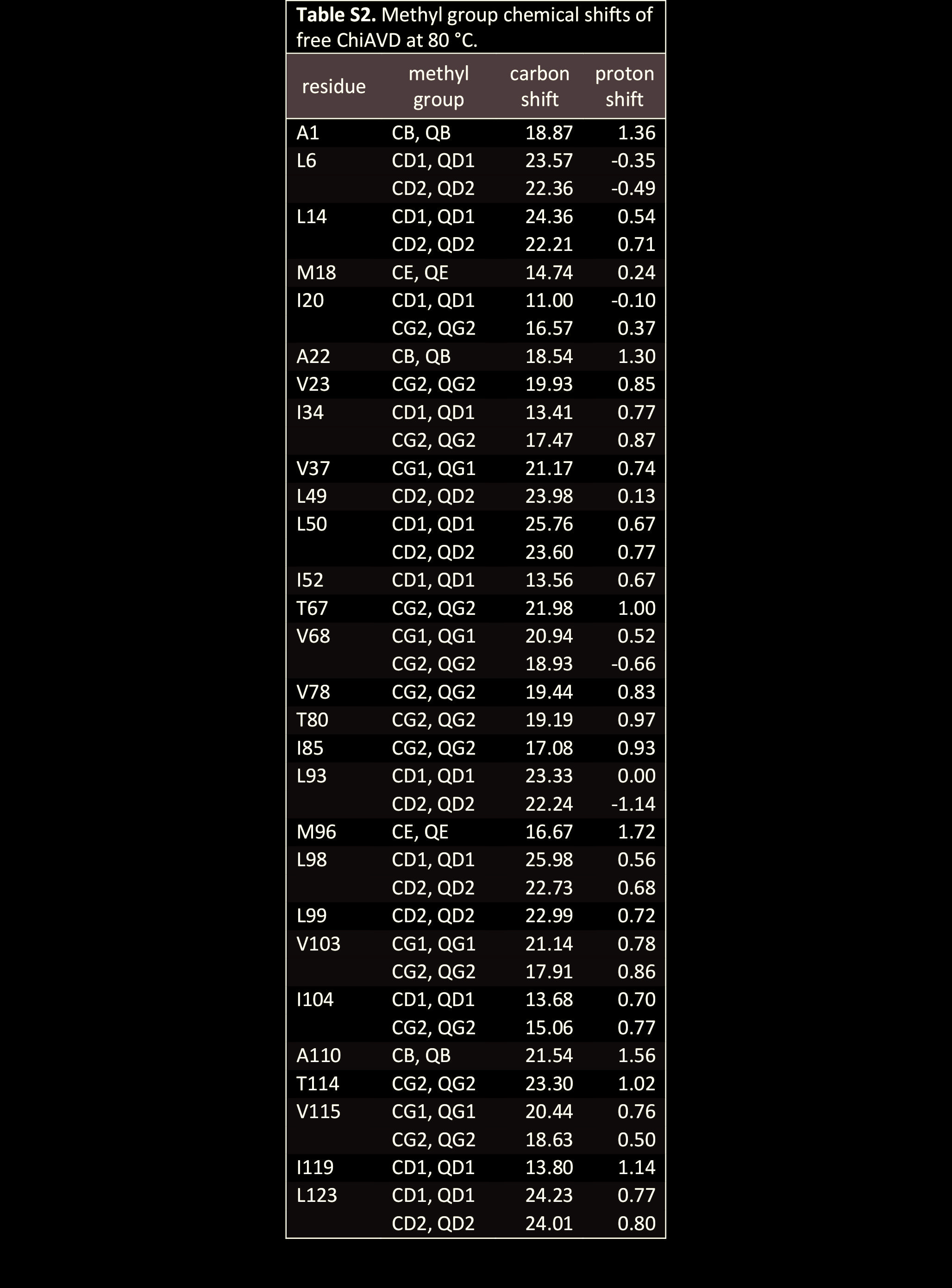

Supplement: Table S2 — Methyl group chemical shifts of free ChiAVD at 80°C. (TIF) [file pone.0100564.s007.tif]

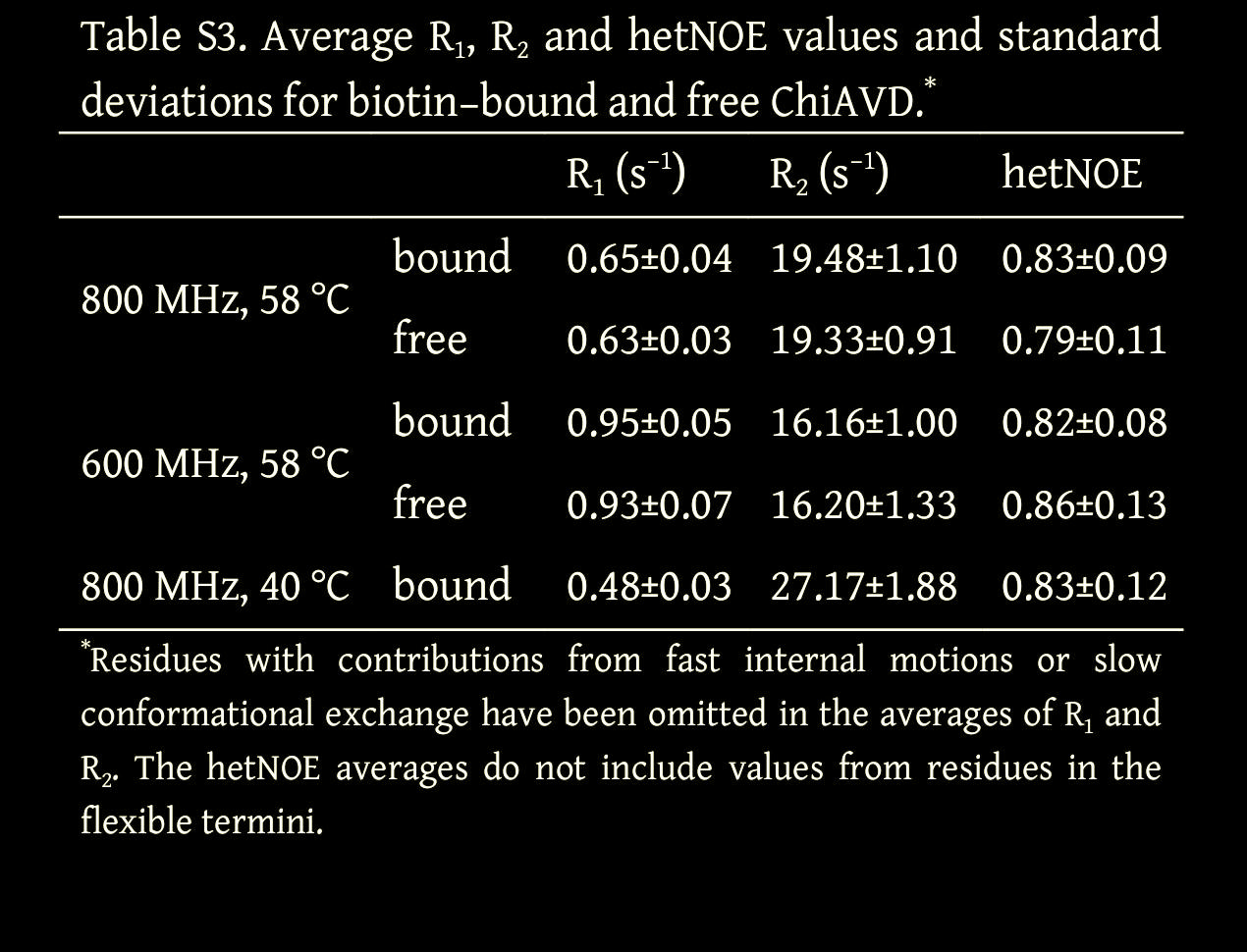

Supplement: Table S3 — Average R1, R2 and hetNOE values and standard deviations for biotin–bound and free ChiAVD. (TIF) [file pone.0100564.s008.tif]
